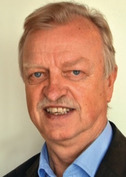# Efficient Aerobic Oxidation of Organic Molecules by Multistep Electron Transfer

**DOI:** 10.1002/anie.202012707

**Published:** 2021-03-24

**Authors:** Jie Liu, Arnar Guðmundsson, Jan‐E. Bäckvall

**Affiliations:** ^1^ State Key Laboratory of Chemo/Biosensing and Chemometrics College of Chemistry and Chemical Engineering Hunan University 410082 Changsha China; ^2^ Department of Organic Chemistry Arrhenius Laboratory Stockholm University SE-10691 Stockholm Sweden; ^3^ Department of Natural Sciences Mid Sweden University Holmgatan 10 SE-85170 Sundsvall Sweden

**Keywords:** electron transfer mediator, green oxidation, homogenous catalysis, mechanisms, molecular oxygen

## Abstract

This Minireview presents recent important homogenous aerobic oxidative reactions which are assisted by electron transfer mediators (ETMs). Compared with direct oxidation by molecular oxygen (O_2_), the use of a coupled catalyst system with ETMs leads to a lower overall energy barrier via stepwise electron transfer. This cooperative catalytic process significantly facilitates the transport of electrons from the reduced form of the substrate‐selective redox catalyst (SSRC^red^) to O_2_, thereby increasing the efficiency of the aerobic oxidation. In this Minireview, we have summarized the advances accomplished in recent years in transition‐metal‐catalyzed as well as metal‐free aerobic oxidations of organic molecules in the presence of ETMs. In addition, the recent progress of photochemical and electrochemical oxidative functionalization using ETMs and O_2_ as the terminal oxidant is also highlighted. Furthermore, the mechanisms of these transformations are showcased.

## Graphical abstract

Recent progress in aerobic oxidations using electron transfer mediators (ETMs) via multistep electron relay is highlighted. The coupled catalytic systems significantly facilitate the transport of the electrons from the reduced substrate‐selective redox catalyst (SSRC) to O_2_, thereby increasing the efficiency of the aerobic oxidation. This strategy has become powerful and valuable in synthetic organic chemistry.

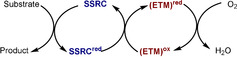

## 1 Introduction

The development of economic and environmentally benign synthetic methods has recently become an important but challenging goal in organic chemistry.^[1]^ Oxidation reactions are involved in a broad range of chemical transformations that allow the facile preparation of various useful commodity chemicals and building blocks.^[2]^ However, in most cases stoichiometric oxidants such as iodosylbenzene, hypochlorite, persulfate, and metal salts (Cu^II^, Ag^I^, Cr^VI^, Mn^VII^) are used, which leads to low atom economy, and considerable amounts of undesired waste. Among various oxidants, molecular oxygen (O_2_) and hydrogen peroxide (H_2_O_2_) are environmentally friendly and highly atom‐efficient oxidants that generate no toxic byproducts.^[3]^ Compared to H_2_O_2_, O_2_ is much less expensive since air can often be used, but it has the disadvantage that rigorous precautions are required for large‐scale applications, especially for high pressure reactions.

**Scheme 1:**
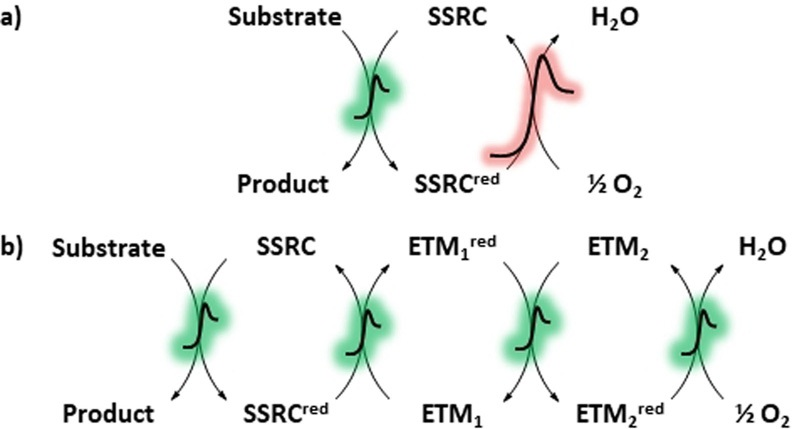
Aerobic oxidation of organic molecules with electron transfer mediators.

Over the past decades, homogenous catalytic aerobic oxidations have provided the basis for the streamlined conversion of various feedstocks into valuable products in industry.^[4]^ However, the direct reoxidation of the reduced form of the substrate‐selective redox catalyst (SSRC^red^) by O_2_ is always challenging due to the often high energy barrier of this reaction step, as well as its low efficiency and selectivity (Scheme 1 a).^[5]^ This “oxidation problem” can be explained by the slow electron transfer between SSRC^red^ and O_2_, compared to the fast deactivation of the catalyst.

One elegant strategy is to mimic biological oxidation systems and insert electron transfer mediators (ETMs) between the substrate‐selective redox catalyst and O_2_ (Scheme 1 b).^[6]^ It is widely recognized that nature's elegant way of overcoming the “oxidation problem” is through the orchestration of a variety of enzymes and co‐enzymes that act as catalysts and ETMs, which lower the overall barrier for electron transfer from SSRC^red^ to O_2_.^[7]^ These ETMs are a part of what is called the electron transport chain (ETC), where O_2_ is typically used as the terminal oxidant. This chain bypasses the high kinetic barrier associated with the direct oxidation of SSRC^red^ by O_2_ and leads to a lower overall energy barrier via stepwise electron transfer.

One of the first examples of the use of an ETM in an aerobic oxidation was the Wacker process. Cupric chloride was used as an ETM to facilitate electron transfer between Pd^0^ and O_2_.^[8]^ A nature‐inspired aerobic oxidative reaction using two ETMs was reported by the Bäckvall group in the late 1980s.^[9]^ Later on, several groups explored this concept by developing mild and efficient coupled catalyst systems with ETMs in various oxidative functionalizations.^[10]^ In general, a good candidate ETM in a redox reaction should have the appropriate redox potential, lead to a low energy barrier in the electron transfer, be stable enough under the reaction conditions, and be compatible with the redox processes involved. In 2008, we summarized catalytic oxidations with O_2_ and H_2_O_2_ as terminal oxidants in multistep electron transfer processes,^[11]^ and a related review was also published by Chang in 2004.^[12]^ After the publication of these reviews, considerable advances have been made in this field.

**Scheme 2:**
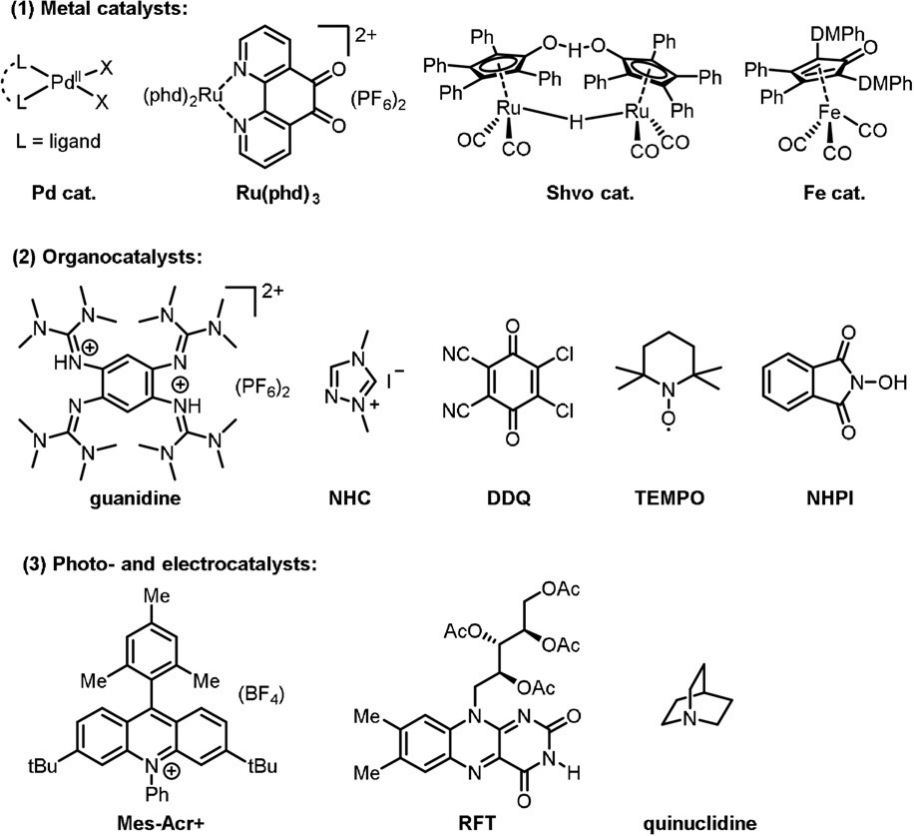
Representative redox catalysts for aerobic oxidation in this Minireview.

Herein we provide an overview of the recent advances in aerobic oxidations assisted by ETMs. In this Minireview, we have selected recent representative examples of aerobic oxidation of organic molecules with redox catalysts, which are divided into three sections (Scheme 2): 1) transition‐metal‐catalyzed aerobic oxidations, using metal catalysts based on palladium, ruthenium, and iron; 2) organocatalyzed aerobic oxidations, using organocatalysts such as guanidine, NHC, DDQ, and nitroxyl; and 3) recent progress in photo‐ and electrocatalyzed oxidative functionalizations. It should be noted that there are also systems where the catalyst is oxidized directly by O_2_.^[13]^ However, this strategy always requires higher reaction temperature, ancillary ligands, or external additives to facilitate the direct oxidation. Coupled catalytic systems with ETMs constitute an important complement to homogenous oxidation chemistry, which has greatly expanded the use of O_2_ as a green oxidant. We hope this Minireview will be of value to chemists involved in oxidation reactions in both academic and industrial research and that it will stimulate further development in green and sustainable chemistry.

## 2 Metal‐Catalyzed Aerobic Oxidation with ETMs

### 2.1 Palladium‐Catalyzed Oxidations (SSRC=Pd)

**Scheme 3:**
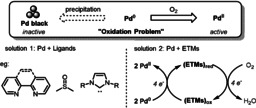
Oxidation problem in Pd‐catalyzed aerobic reactions and the solutions.

Palladium‐catalyzed aerobic oxidations have emerged as powerful and valuable tools in modern organic synthesis. Despite significant advances in this field, a severe problem often encountered is the fast aggregation of palladium black from participating palladium species (Pd‐H or Pd^0^) which slows down and finally stops the homogenous reaction (Scheme 3). Extensive endeavors to solve this problem have focused on the use of air‐stable ligands such as sulfoxides, amines, pyridines, and carbenes, to prevent Pd^0^ precipitation during the catalytic cycle. Stahl and co‐workers published a review on the use of various ligands that improve catalyst efficiency and stability in aerobic oxidations with increased selectivity.^[14]^ On the other hand, coupled catalytic systems with an ETM can facilitate the relay of electrons between the Pd catalyst and O_2_. These coupled catalytic systems have been demonstrated to be highly efficient in Wacker oxidations, allylic oxidations, C‐H activations, and carbocyclizations.

**Scheme 4:**
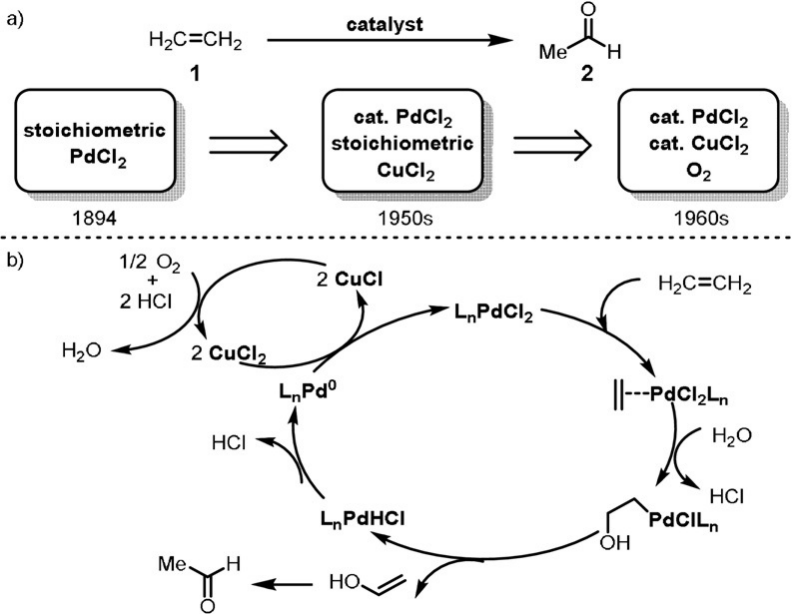
a) Overview of the history of Wacker oxidation; b) General mechanism of the Wacker oxidation of ethylene to acetaldehyde.

The Pd‐catalyzed Wacker–Tsuji reaction is one of the most famous homogenous reactions which provides reliable access to carbonyl compounds (aldehydes or ketones) under mild reaction conditions.^[15]^ This reaction refers generally to the transformation of a terminal or 1,2‐disubstituted olefin to an aldehyde or ketone, respectively, through the action of Pd^II^, water, and a co‐oxidant. Conversion of ethylene to acetaldehyde by stoichiometric PdCl_2_ was discovered over a century ago;^[16]^ however, it was not until sixty years later that a catalytic method was developed. In the 1950s, researchers at Wacker Chemie reported that a transformation took place in an aqueous, acidic solution of catalytic PdCl_2_ and a stoichiometric amount of CuCl_2_ through which oxygen was bubbled.^[17]^ Later on, in the 1960s Farbwerke Hoechst developed a one‐stage process using oxygen as the terminal oxidizing agent (Scheme 4).^[18]^ This process quickly attracted great interest around the world since acetaldehyde is an important intermediate in industrial chemistry, with over two million tons being produced annually today.

To encourage sufficient mixing of the organic reactants with the aqueous phase, a co‐solvent is generally employed along with water. When dimethylformamide (DMF) is used as a co‐solvent with CuCl_2_ as the ETM under atmospheric pressure of oxygen, the reaction is called the “Tsuji–Wacker oxidation”.^[19]^ This version allows a range of olefins, which are not soluble in water, to be used. The mechanism of the Wacker oxidation has been studied for over 60 years with one of the earliest studies being reported by Smidt et al.[^15c^, ^20^] The generally accepted mechanism is the one in which an alkene coordinates to Pd^II^ with subsequent nucleophilic attack by water and β‐hydride elimination to afford the carbonyl product. Conventionally, CuCl_2_ and O_2_ are used as the oxidants to regenerate Pd^II^ from the Pd^0^ formed.

**Scheme 5:**
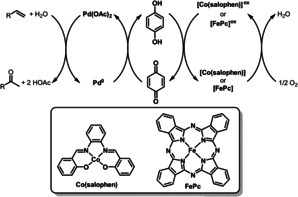
Chloride‐free Pd‐catalyzed Wacker‐type oxidations.

**Scheme 6:**
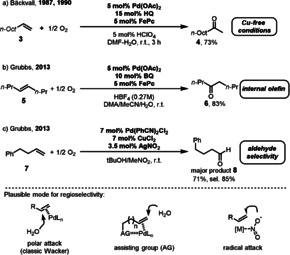
Representative Wacker‐type reactions and the key intermediates for regioselectivity.

Despite the success of the Wacker reaction, the presence of chloride ions has a negative effect on the reaction efficiency and causes selectivity problems through the formation of chlorinated side products. To solve these problems associated with the use of chloride, Bäckvall reported a chloride‐free Wacker oxidation by using Pd(OAc)_2_ together with hydroquinone (HQ) and a metal macrocycle, such as a cobalt Schiff base complex (Co(salophen)) and iron phthalocyanine (FePc) as ETMs (Scheme 5).^[21]^ This triple catalytic system has led to a faster and more efficient reaction, giving high yields under mild conditions with 5 mol % Pd catalyst as shown in Scheme 6 a. This catalytic procedure is approximately 16 times faster than the corresponding chloride‐based Wacker‐type oxidation.^[22]^

However, Bäckvall's reaction was limited to the oxidation of terminal olefins. This is because internal olefins show relatively low reactivity and selectivity. Kaneda and co‐workers developed a copper‐free Wacker oxidation of internal olefins.^[23]^ This protocol requires the use of relative high oxygen pressures (3–9 atm) in an autoclave. In 2013, Grubbs developed a general and practical Pd‐catalyzed oxidation to access ketones from a wide variety of internal olefins (Scheme 6 b).^[24]^ The use of HBF_4_ as an acid additive in the DMA/MeCN/H_2_O solvent mixture resulted in high conversion of internal olefin **5** to the corresponding ketone **6**. It is likely that a dicationic complex is generated in situ in the presence of HBF_4_ through protonation of the acetate ligands. This process showed wide functional‐group tolerance for the preparation of ketones with a triple catalytic system using O_2_ as the terminal oxidant.

Another key issue in the Wacker oxidation is the regioselectivity of the product.^[25]^ The majority of terminal alkenes produce predominately methyl ketones, which is in accordance with Markovnikov's rule. Feringa reported that anti‐Markovnikov selectivity could be achieved through the Pd‐catalyzed oxidation of ester‐ or amide‐protected allylic substrates using BQ as an oxidant.^[26]^ The regioselectivity can be explained by the weak coordinating influence of the Lewis basic oxygen or nitrogen functional groups. In 2013, Grubbs developed a nitrite‐modified Wacker‐type catalyst system for the conversion of simple aliphatic alkenes **7** to aldehydes **8** (Scheme 6 c).^[27]^ The addition of a nitrite source, such as AgNO_2_, led to good anti‐Markovnikov selectivity and synthetically useful yields. It is proposed that the catalyst facilitates in situ formation of an NO_2_ radical from the nitrite salt, followed by a radical‐type addition of the NO_2_ radical to the olefin at the terminal position. This determines the ultimate origin of anti‐Markovnikov selectivity.

**Scheme 7:**
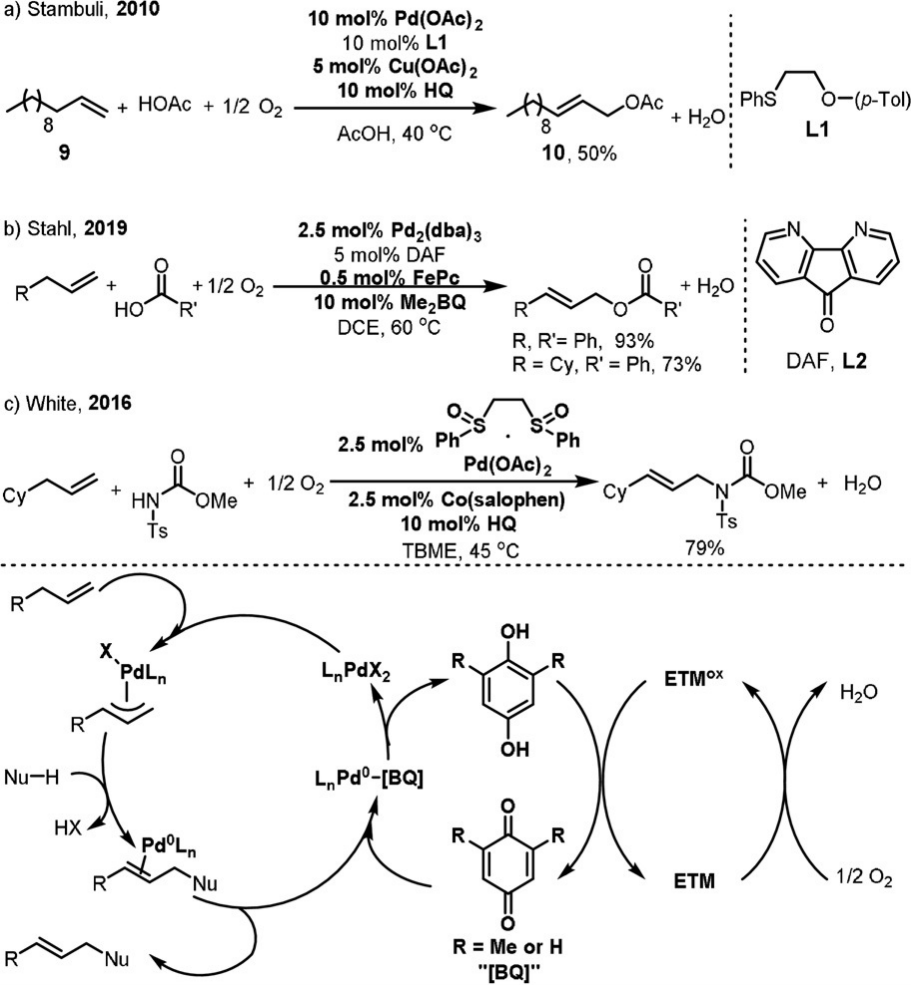
Pd‐catalyzed allylic C‐H functionalizations.

Palladium‐catalyzed allylic C‐H activation represents a powerful synthetic strategy in organic chemistry.^[28]^ In 1990, the Bäckvall group reported a coupled quinone/metal macrocycle system for the aerobic allylic acetoxylation of olefins.^[21b]^ This reaction is efficient and gives the allylic acetate in good yield through the oxidation of cyclohexene under atmospheric O_2_. However, only acetic acid was applied as the coupling partner in this reaction. In 2010, Stambuli showed that the sulfide ligand **L1** can improve the allylic acetoxylation of terminal olefins **9** when O_2_ is used in combination with 5 mol % Cu(OAc)_2_ (Scheme 7 a).^[29]^ Also, in this reaction only acetic acid was applied as the coupling partner. A method compatible with diverse carboxylic acids was developed by the Stahl group (Scheme 7 b).^[30]^ The use of 4,5‐diazafluoren‐9‐one (DAF, **L2**) as a ligand, in combination with a quinone/FePc co‐catalyst system leads to efficient oxidation of Pd^0^ by O_2_ which then promotes acyloxylation of the allylic C−H bond. A more sterically hindered 2,6‐dimethylbenzoquinone (Me_2_BQ) was found to improve the product yield. The quinone is not only a co‐catalyst for transporting electrons between the Pd catalyst and the metal macrocycle, but can also act as a ligand, which coordinates to the Pd species during the catalysis.^[31]^ In addition to C‐O couplings, Pd‐catalyzed allylic C‐H amination was reported by the White group in 2016 (Scheme 7 c).^[32]^ The use of redox‐active co‐catalysts, Co(salophen), and HQ as ETMs significantly improved the reaction efficiency under atmospheric O_2_.

**Scheme 8:**
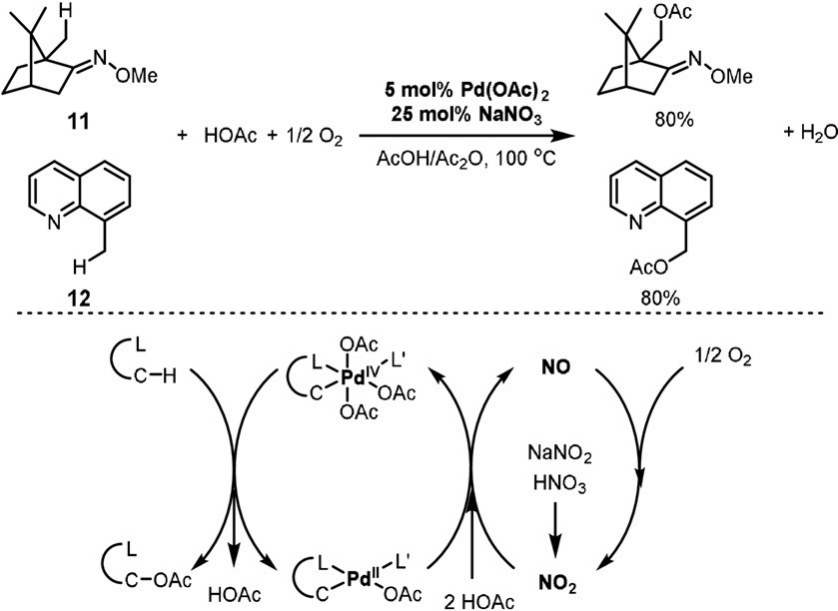
Pd‐catalyzed aerobic C(sp^3^)‐H acetoxylation.

An example of aerobic C(sp^3^)−H activation was reported by the Sanford group in 2012, which focused on the use of NaNO_3_ or NaNO_2_ as an ETM in a Pd‐catalyzed aerobic oxidation of unactivated C(sp^3^)−H bonds using oxime ethers **11** or quinoline derivatives **12** as effective directing groups (Scheme 8).^[33]^ This process proceeds through the decomposition of nitrate to NO_2_ which is the active ETM. The presence of NO was confirmed through the addition of butylated hydroxytoluene (BHT) to the reaction, which is known to form 2,6‐di‐*tert*‐butyl‐4‐methyl‐4‐nitrosocyclohexa‐2,5‐dienone (TBMND) on exposure to NO.

**Scheme 9:**
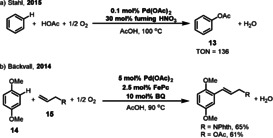
Pd‐catalyzed aerobic functionalization of aromatic C−H bond.

The oxidative functionalization of simple aromatic C−H bonds was originally reported by Fujiwara and Moritani.^[34]^ Much progress has been achieved in this field following this pioneering work, which is now recognized as a powerful method for the construction of valuable building blocks.^[35]^ The Stahl group described the use of nitrite and nitrate sources (fuming HNO_3_) as NO_*x*_‐based redox mediators in the acetoxylation of benzene (Scheme 9 a).^[36]^ These reaction conditions avoid the formation of biphenyl as a side product, and strongly favor formation of phenyl acetate **13** over nitrobenzene (selectivity PhOAc:PhNO_2_ up to 40:1). Under the optimized reaction conditions, with 0.1 mol % Pd(OAc)_2_, 136 turnovers of the Pd catalyst are achieved with only 1 atm of O_2_ pressure. The Bäckvall group reported an efficient Pd‐catalyzed aerobic oxidative dehydrogenative coupling between arene **14** and nonbiased olefin **15** (Scheme 9 b).^[37]^ This reaction took place in the presence of catalytic amounts of BQ and FePc as the ETMs under ambient oxygen pressure.

**Scheme 10:**
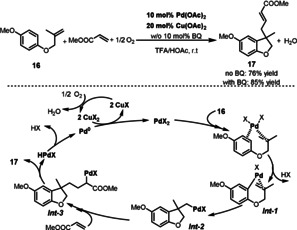
Pd‐catalyzed carbocyclization to dihydrobenzofurans.

In 2017, Jiang and Li described a Pd‐catalyzed cascade oxidative arylation/olefination for the synthesis of dihydrobenzofurans **17** (Scheme 10).^[38]^ Similar to Wacker‐type oxidations, the use of a catalytic amount of Cu(OAc)_2_ as the ETM allows efficient reoxidation of the Pd catalyst under aerobic conditions. In this reaction, the addition of BQ increased the yield from 76 % to 85 %. This result can be explained by the fact that BQ stabilizes the Pd^0^ species through the in situ generation of Pd^0^‐BQ complexes, and no palladium black was observed. The mechanism of the reaction involves electrophilic metalation of substrate **16** by PdX_2_ (X=OTFA or OAc) to give aryl‐Pd species ***Int‐1***. Then, intramolecular insertion of the olefin forms the alkyl‐Pd intermediate ***Int‐2***, which is followed by insertion of activated olefin to give ***Int‐3***. A β‐hydride elimination affords the corresponding product **17** and the active Pd^II^ species is regenerated through oxidation of Pd^0^ by CuX_2_/O_2_.

**Scheme 11:**
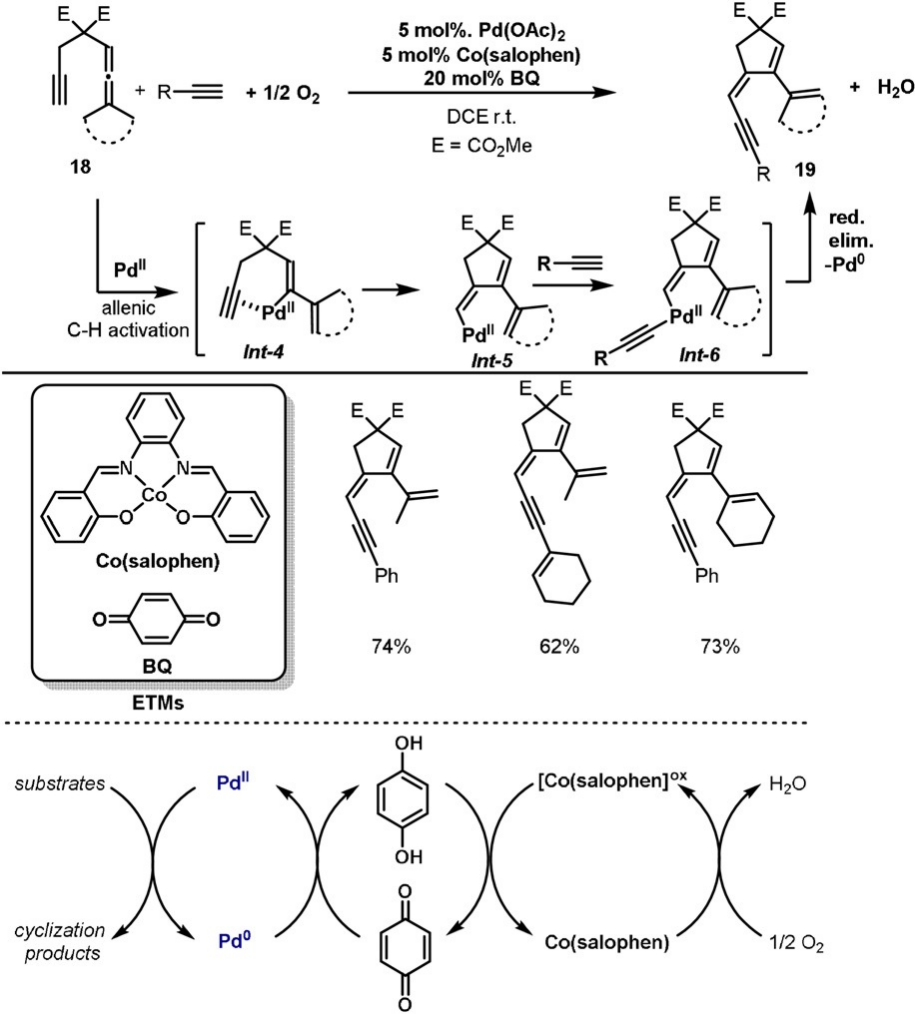
Pd‐catalyzed aerobic carbocyclization of allenynes.

Pd‐catalyzed oxidative carbocyclizations have become powerful methods for the construction of carbocyclic and heterocyclic compounds.^[39]^ The Bäckvall group reported an aerobic catalyst system for the oxidative carbocyclization of allenyne **18** to five‐membered carbocycle **19** (Scheme 11).^[40]^ Initially, the coordination of the allene and alkyne units to the Pd^II^ center leads to an allenic C(sp^3^)–H bond cleavage and the generation of a vinylpalladium intermediate ***Int‐4***. Carbocyclization of ***Int‐4*** via alkyne insertion gives ***Int‐5*** and reaction with the free alkyne generates ***Int‐6***. Reductive elimination from ***Int‐6*** provides the target product **19** and a Pd^0^ species. Interestingly, there was no competition between the two terminal alkynes, and the five‐membered carbocycle was obtained in high selectivity. With the assistance of BQ and Co(salophen) as ETMs, aerobic oxidation of Pd^0^ regenerates Pd^II^. Different substituted allenynes were subjected to the reaction conditions and gave the corresponding five‐membered carbocycles in good yields and high stereoselectivity.

**Scheme 12:**
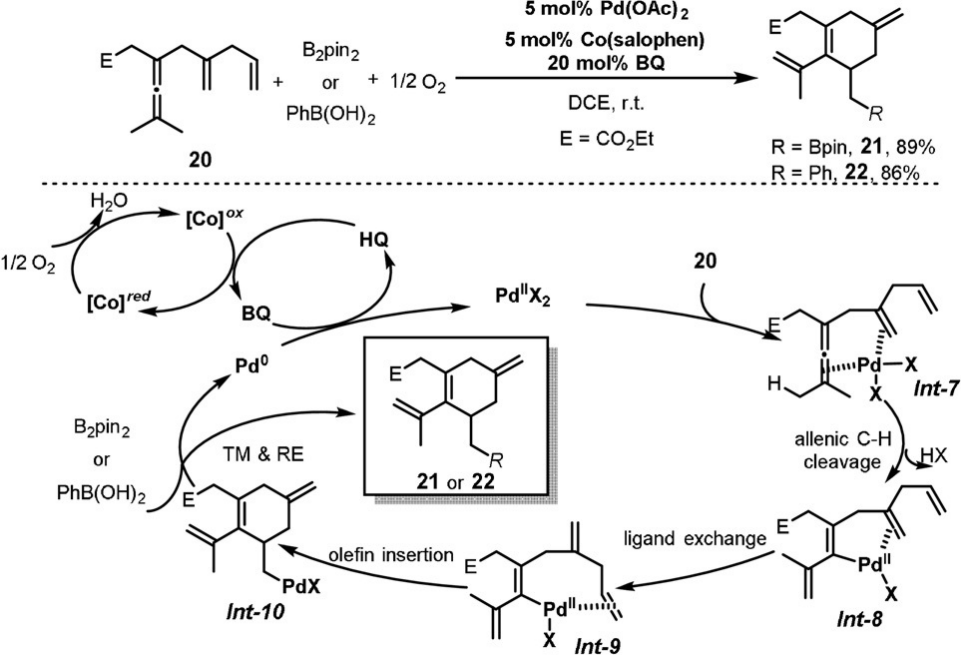
Pd‐catalyzed aerobic carbocyclization of dienallenes.

This catalyst was also applied for a selective olefin‐assisted Pd‐catalyzed oxidative carbocyclization of enallene **20** via remote olefin insertion to afford substituted cyclohexenes **21** and **22** (Scheme 12).^[41]^ The reaction of Pd^II^ with enallene **20** bearing the assisting olefin produces vinylpalladium ***Int‐8*** via allenic C−H bond cleavage, which is promoted by the coordination of the allene and the assisting olefin to Pd^II^ in ***Int‐7***. Then, the vinylpalladium intermediate ***Int‐9*** would be generated from ***Int‐8*** via ligand exchange (from proximal olefin to remote olefin). Next, ***Int‐9*** would undergo a remote olefin insertion to give cyclic intermediate ***Int‐10***. Subsequent transmetalation of ***Int‐10*** with B_2_pin_2_ or arylboronic acid, followed by reductive elimination would give the target cyclohexene derivatives **21** or **22**. The biomimetic approach using catalytic amounts of Co(salophen) and BQ as the ETMs led to aerobic borylative and arylative carbocyclizations in good yields.

**Scheme 13:**
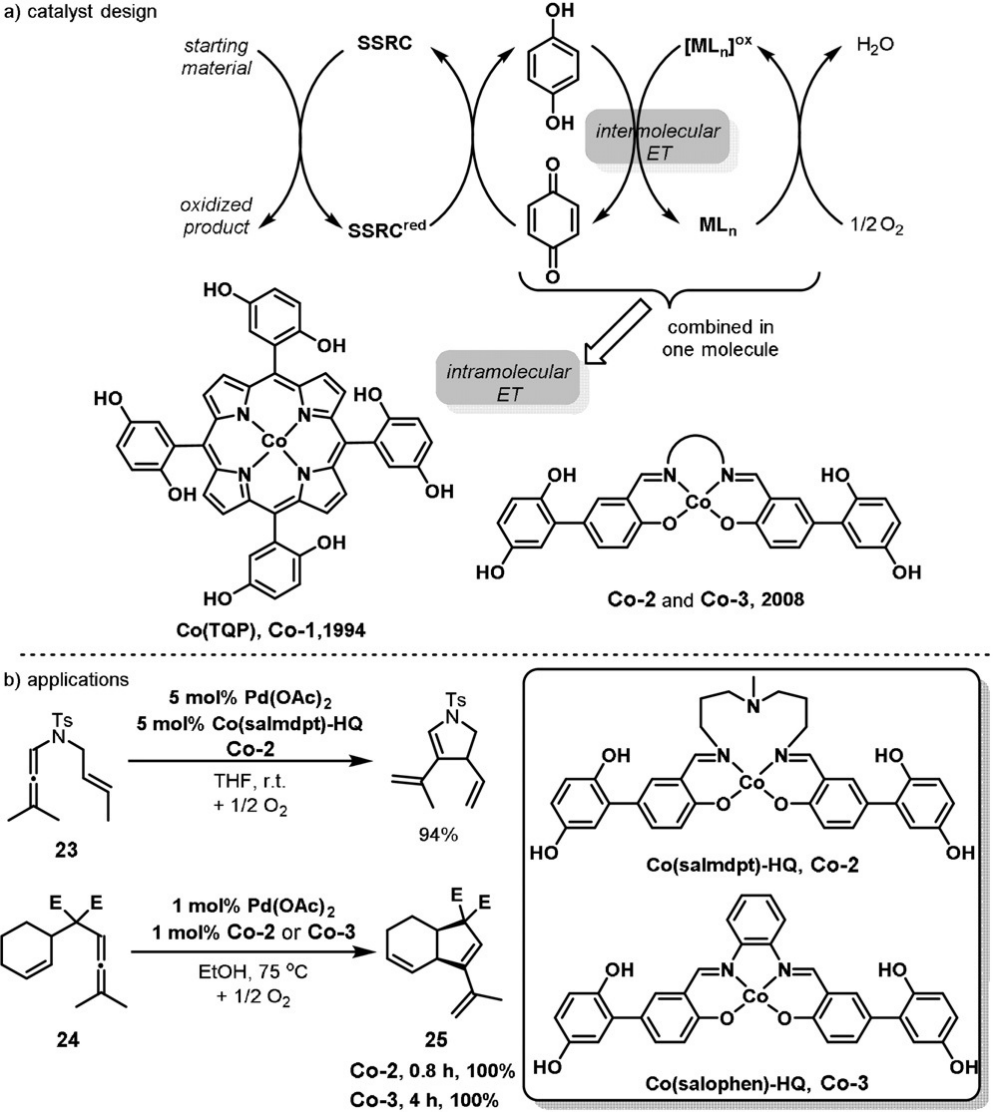
a) Design of a bifunctional hybrid ETM for aerobic oxidations; b) Applications in the Pd‐catalyzed carbocyclization of enallenes.

In addition to the previous catalyst system with the quinone and metal macrocycle as separate ETMs, a homogenous hybrid catalyst was developed by Bäckvall, in which the metal macrocycle and quinone are merged into one molecule (Scheme 13 a).[^42^, ^43^] In 1993, Bäckvall developed an efficient hybrid catalyst (Co(TQP), **Co‐1**), involving a cobalt‐porphyrin with pendant hydroquinone groups in one molecule for aerobic Pd‐catalyzed 1,4‐diacetoxylation of 1,3‐cyclohexadiene, but the stereoselectivity of the reaction was moderate and the catalyst was difficult to synthesize. In 2008, the same group designed an improved hybrid Schiff base–hydroquinone as a redox relay catalyst for aerobic oxidations.^[43]^ In this type of hybrid catalyst, cobalt Schiff base complexes were chosen as the oxygen‐activating center because of their demonstrated efficiency in coupled aerobic oxidation, and their simple and modular synthesis. For example, the hybrid cobalt catalyst **Co‐2** Co(salmdpt)‐HQ (salmdptH_2_=bis[3(salicylideneimino)propyl]methylamine), and **Co‐3** Co(salophen)‐HQ were used as efficient ETMs in the Pd‐catalyzed aerobic oxidative carbocyclizations of enallene **23** and **24** (Scheme 13 b).^[44]^ The authors mentioned that the slower reaction of **24** to **25** with **Co‐3** is most likely due to the fact that **Co‐3** is quite insoluble in ethanol.

**Scheme 14:**
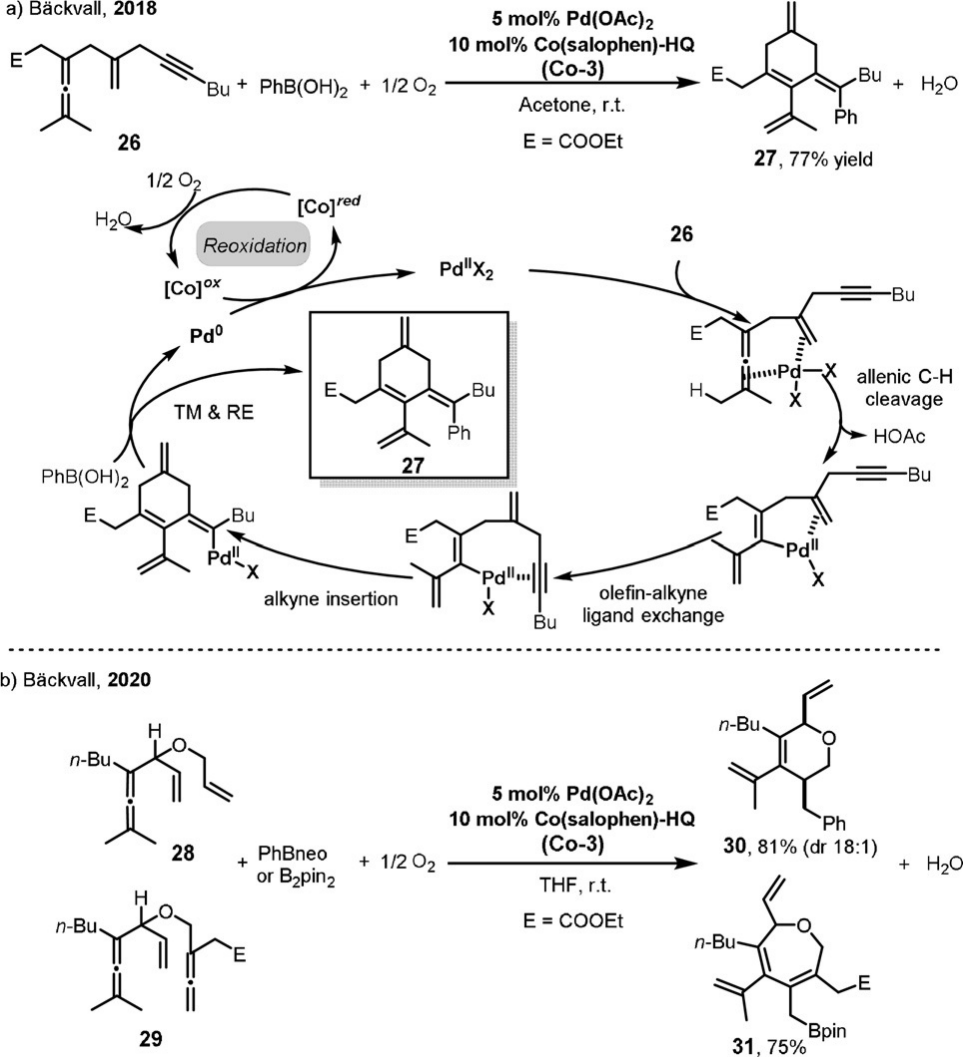
a) Catalytic cycle of Pd‐catalyzed aerobic oxidative carbocyclization of enallenynes; b) Pd‐catalyzed aerobic oxidative carbocyclization of dienallenes and bisallenes.

The Co(salophen)‐HQ **Co‐3** was also found to be highly efficient in the Pd‐catalyzed aerobic carbocyclization of the rationally designed enallenyne **26** (Scheme 14 a).^[45]^ The enallenyne **26** contains three different C−C π‐bond functionalities (allene, olefin, and alkyne groups), and this combination presents a challenge concerning the control of regioselectivity in the reaction. Interestingly, cyclization takes place between the allene moiety and the distal triple bond of the enallenyne **26**, while the olefin group remains intact. The mechanism is similar to that of the carbocyclization of enallene **20**, in which a “helping olefin” triggers the C(sp^3^)−H bond cleavage followed by a replacement of the coordinated pendant olefin by the remote unsaturated C‐C ligand. Control experiments showed that the initial step of the allenic C(sp^3^)–H bond cleavage by Pd^II^ requires the coordination of a pendant olefin bond in the substrate. The use of **Co‐3** resulted in a significantly higher reaction rate than that with Co(salophen) and quinone as separate ETMs. The authors proposed that the hybrid catalyst, **Co‐3** enhances the overall electron transfer between the Pd catalyst and O_2_, due to intramolecular electron transfer in **Co‐3**, thereby leading to a more efficient overall reaction under aerobic conditions. In addition, the catalyst **Co‐3** is also highly efficient in the oxidative carbocyclization of dienallenes **28** and bisallenes **29** to six‐ or seven‐membered heterocycles **30** and **31** (Scheme 14 b).^[46]^

### 2.2 Ruthenium‐ and Iron‐Catalyzed Oxidations (SSRC=Ru or Fe)

**Scheme 15:**
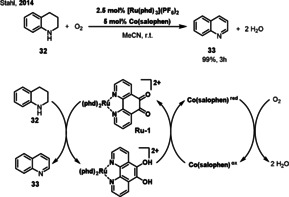
Ru‐catalyzed aerobic dehydrogenations of tetrahydroquinolines.

Ruthenium‐catalyzed oxidation reactions are a class of important transformations in organic synthesis.^[47]^ In the past decades, mild dehydrogenations of alcohols to give aldehydes or ketones through the use of low‐valent Ru complexes have been reported, which were subsequently extended to the dehydrogenation of amines to imines.^[48]^ The Stahl group reported a Ru‐based catalyst system that shows high activity for amine oxidation, including the successful aerobic dehydrogenation of diverse tetrahydroquinolines at room temperature with ambient air (Scheme 15).^[49]^ In this work, the ruthenium complex [Ru(phd)_3_]^2+^ (**Ru‐1**, phd=1,10‐phenanthroline‐5,6‐dione) was applied for the oxidative dehydrogenation of tetrahydroquinoline **32** to afford quinoline **33**. The structure of [Ru(phd)_3_](ClO_4_)_2_ was characterized via X‐ray crystallography. The use of a Co(salophen) as the ETM allows the reaction to proceed efficiently. In the absence of Co(salophen), the reaction is very slow. The synthetic utility of the catalytic method was demonstrated by the preparation of various medicinally relevant quinolines.

**Scheme 16:**
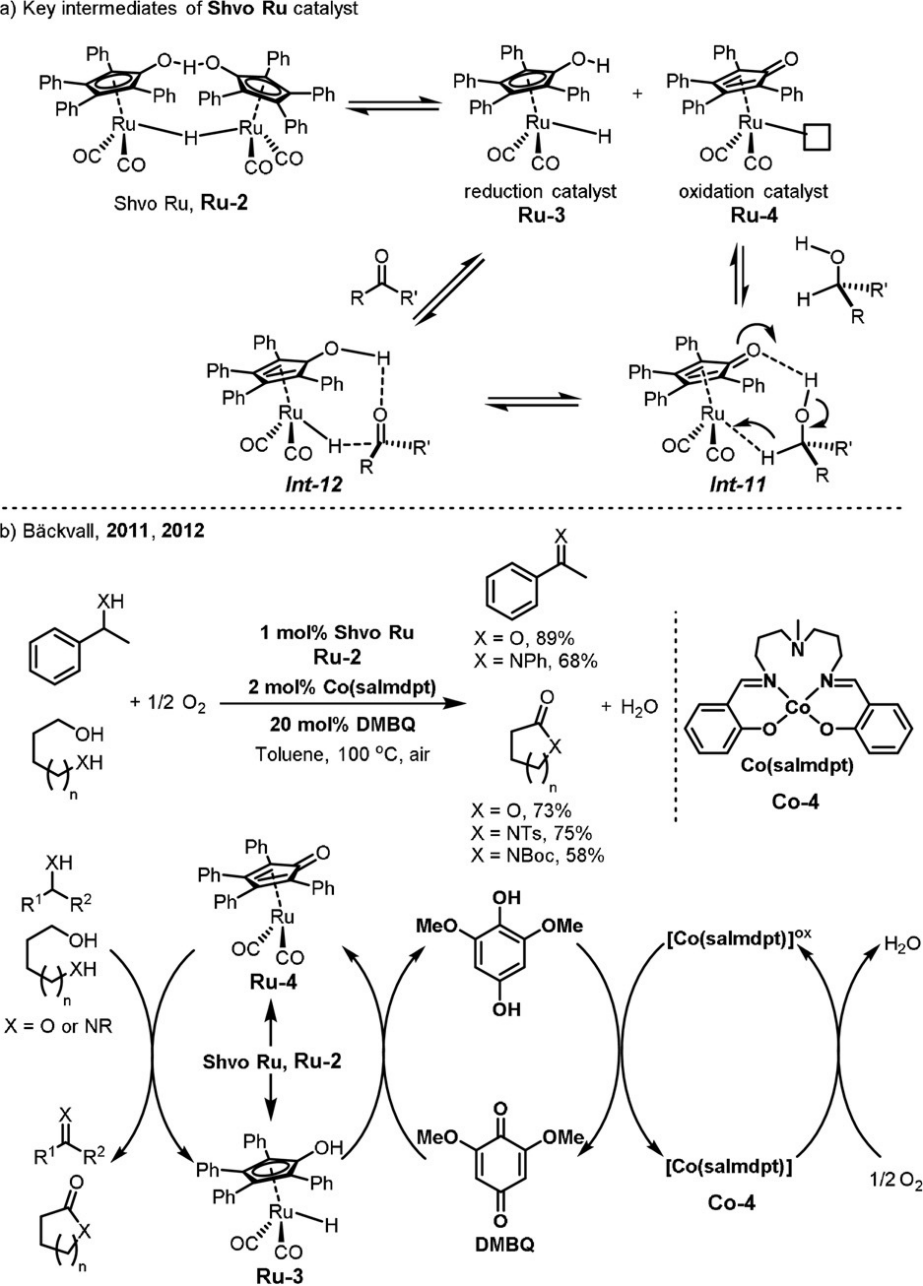
a) Shvo's ruthenium complexes and the mechanism for the hydride transfer process; b) Ru‐catalyzed aerobic dehydrogenation of alcohols and amines.

A dimeric ruthenium catalyst called Shvo catalyst **Ru‐2** has been known since the 1980s and has been demonstrated to be highly efficient in a large variety of hydrogenation and oxidation transformations.^[50]^ The dissociation of **Ru‐2** gives two complementary active monomeric species **Ru‐3** and **Ru‐4** bearing a non‐innocent active functionalized ligand (Scheme 16 a). The hydroxycyclopentadienyl ligand in **Ru‐3** has a proton on the oxygen and the Ru center has a hydride ligand. The proton and the hydride are involved in hydrogen transfer reactions. The cyclopentadienone in **Ru‐4** has a proton‐acceptor site at oxygen and a hydride‐acceptor site at the Ru center and can catalyze oxidations. The dehydrogenation of alcohols occurs through an outer‐sphere pathway in which the alcohol binds to the Ru complex through bridging hydrogens (***Int‐11***).^[51]^ The alcohol complex ***Int‐11*** undergoes simultaneous hydride and proton transfer to produce ***Int‐12***, which finally releases the ketone. The Bäckvall group developed efficient Ru‐catalyzed aerobic oxidations via a biomimetic catalytic system (Scheme 16 b).^[52]^ Dehydrogenation of the alcohol by **Ru‐4** affords the oxidized product and the ruthenium hydride **Ru‐3**. The latter species is reoxidized by O_2_ with the aid of an electron transfer system. The Shvo catalyst (**Ru‐2**) showed excellent activity and a more electron‐rich quinone, 2,6‐dimethoxy‐1,4‐benzoquinone (DMBQ), and Co(salmdpt) complex **Co‐4** were used as the ETMs. The authors speculated that the slow step in the electron transfer chain is the reoxidation of the hydroquinone, and the more electron‐rich 2,6‐dimethoxy‐1,4‐hydroquinone is oxidized faster than the 1,4‐hydroquinone by [Co(salmdpt)]^ox^. The oxidation of alcohols, amines, diols, and amino alcohols could be performed with high selectivity to give the corresponding carbonyl products in generally good yields.

**Scheme 17:**
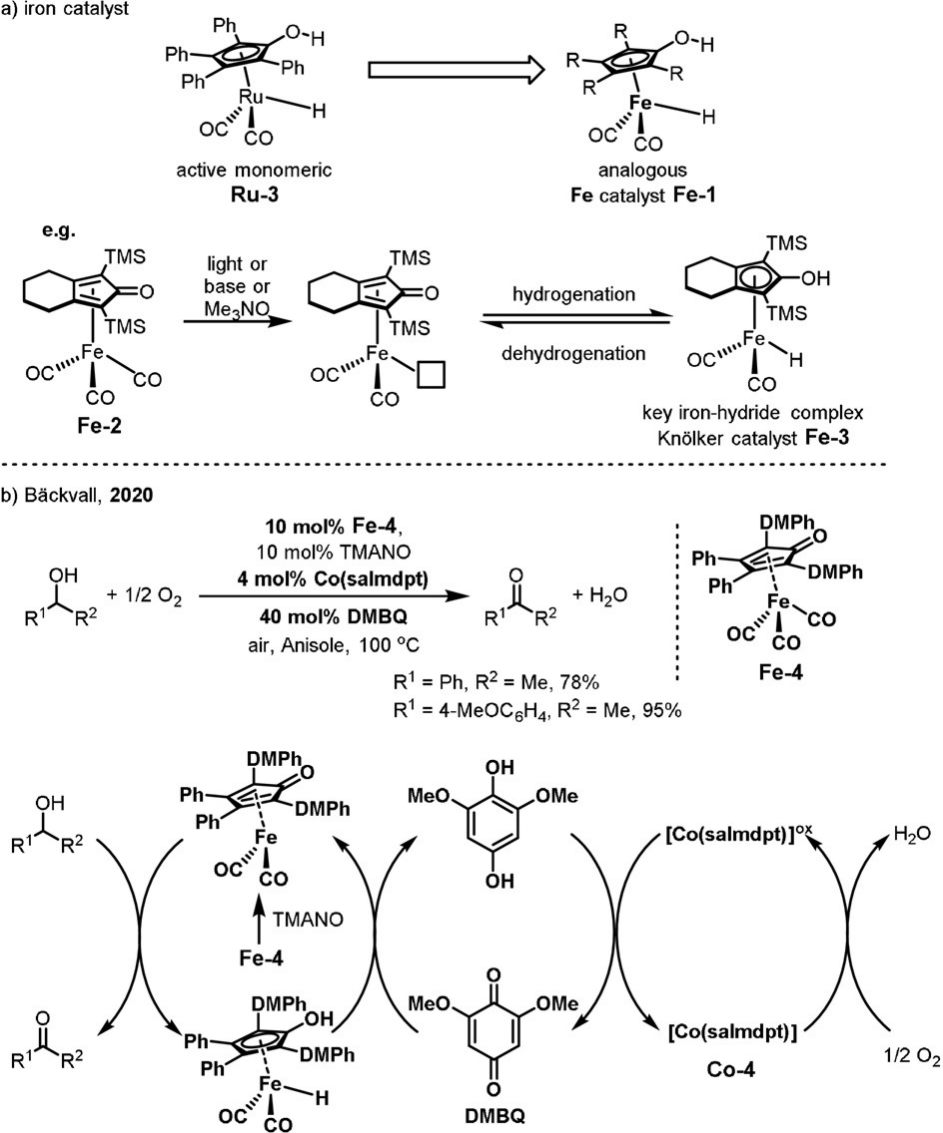
a) Analogy between Ru and Fe hydroxycyclopentadienyl complexes; b) Iron‐catalyzed biomimetic aerobic oxidation of alcohols.

Given the cost‐effective and sustainable nature of earth‐abundant first‐row transition metals, the development of less toxic, inexpensive 3d metal catalysts for aerobic oxidations has gained considerable momentum as a more environmentally friendly and economically attractive alternative.^[53]^ Iron is the most abundant transition metal in the Earth's crust. In addition to the economic benefit, the development of iron‐catalyzed reactions also provides an opportunity to access complementary chemoselectivity and discover new reactivity.^[54]^ Cyclopentadienone iron hydride complexes such as **Fe‐1** (Scheme 17 a) have been known since the 1950s, long before the Shvo precatalyst. In the 1990s, Knölker and co‐workers isolated the first iron hydride hydroxycyclopentadienyl complex **Fe‐3** from the precursor **Fe‐2** and confirmed its structure by X‐ray crystallography.^[55]^ The first catalytic application of hydrogenations and transfer hydrogenations with this type of catalyst was reported by Casey and Guan in 2007.^[56]^ Since then, given their unique catalytic behavior, easy access from abundant iron sources, and good stability, there has been a dramatic increase in the number of catalytic applications using these catalysts.^[57]^ An outer‐sphere mechanism of dehydrogenation analogous to that of the Shvo system was proposed for iron‐catalyzed oxidations.^[58]^ Very recently, Bäckvall reported on an iron‐catalyzed aerobic biomimetic oxidation of alcohols (Scheme 17 b).^[59]^ The electron transfer from the alcohol to O_2_ occurs with the aid of three coupled catalytic redox systems, leading to a low‐energy pathway. In this reaction, the (cyclopentadienone)iron tricarbonyl complex **Fe‐4** was utilized as the substrate‐selective dehydrogenation catalyst along with DMBQ and an oxygen‐activating Co(salmdpt) complex as ETMs. Various primary and secondary alcohols were oxidized in air to their corresponding aldehydes and ketones in good to excellent yields with this method.

### 2.3 Metal‐Free SSRC with Metal‐Containing ETMs

**Scheme 18:**
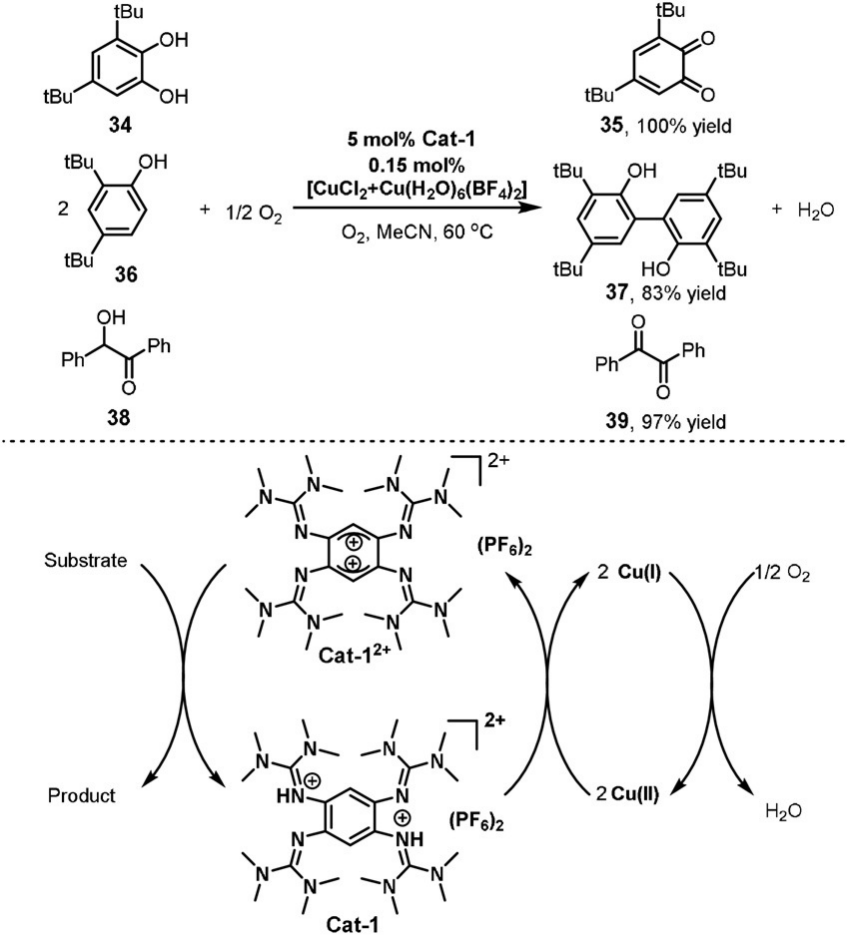
Guanidine‐mediated aerobic oxidations.

In addition to transition‐metal‐catalyzed aerobic oxidations, organic molecules can also act as substrate‐selective redox catalysts for aerobic oxidations. The Himmel group reported a redox‐active guanidine, 1,2,4,5‐tetrakis‐(tetramethylguanidino)benzene **Cat‐1**, that can act as a catalyst in the green aerobic oxidation of organic molecules (Scheme 18).^[60]^ **Cat‐1** is a rather strong electron donor (*E*_1/2_=−0.7 V vs. Fc^+^/Fc for the redox pair **Cat‐1^2+^**/**Cat‐1**). Reduction of the oxidized form, **Cat‐1^2+^**, by proton‐coupled electron transfer is favored by the strong Brønsted basicity of guanidines and the restoration of the aromatic system. This property enables efficient dehydrogenative oxidation of 3,5‐di‐*tert*‐butylcatechol **34** to *ortho*‐benzoquinone **35**, 2,4‐di‐*tert*‐butylphenol **36** to biphenol **37**, and benzoin **38** to benzil **39** in excellent yields. In the other half‐reaction, reoxidation of the guanidine to the oxidized state is achieved with O_2_ in the presence of a copper catalyst.

**Scheme 19:**
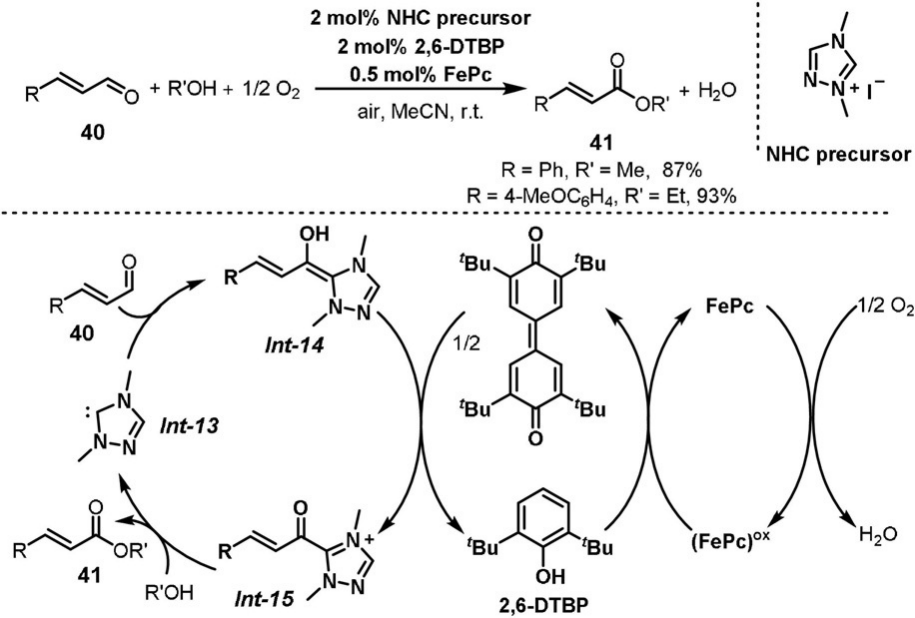
NHC‐catalyzed oxidative esterfication of aldehydes.

The *N*‐heterocyclic carbene (NHC)‐catalyzed aerobic oxidative esterification of aldehydes with alcohols is a green and sustainable process for the synthesis of versatile esters. Sundén and co‐workers described a strategy for oxidative NHC‐catalyzed esterifications of α,β‐unsaturated aldehydes **40** in the presence of FePc and 2,6‐di‐*tert*‐butylphenol (2,6‐DTBP) as ETMs (Scheme 19).^[61]^ In this reaction, the key steps are the oxidation of homoenolate intermediate ***Int‐14*** by quinone (from 2,6‐DTBP) to the acyl azolium ***Int‐15***, which reacts with the alcohol to give the unsaturated ester **41** and regenerating NHC ***Int‐13***. The reaction has a broad substrate scope and the products were isolated in good to excellent yields. The use of air as the terminal oxidant offers an environmentally friendly and inexpensive way to scale up this important oxidation reaction via NHC chemistry.

**Scheme 20:**
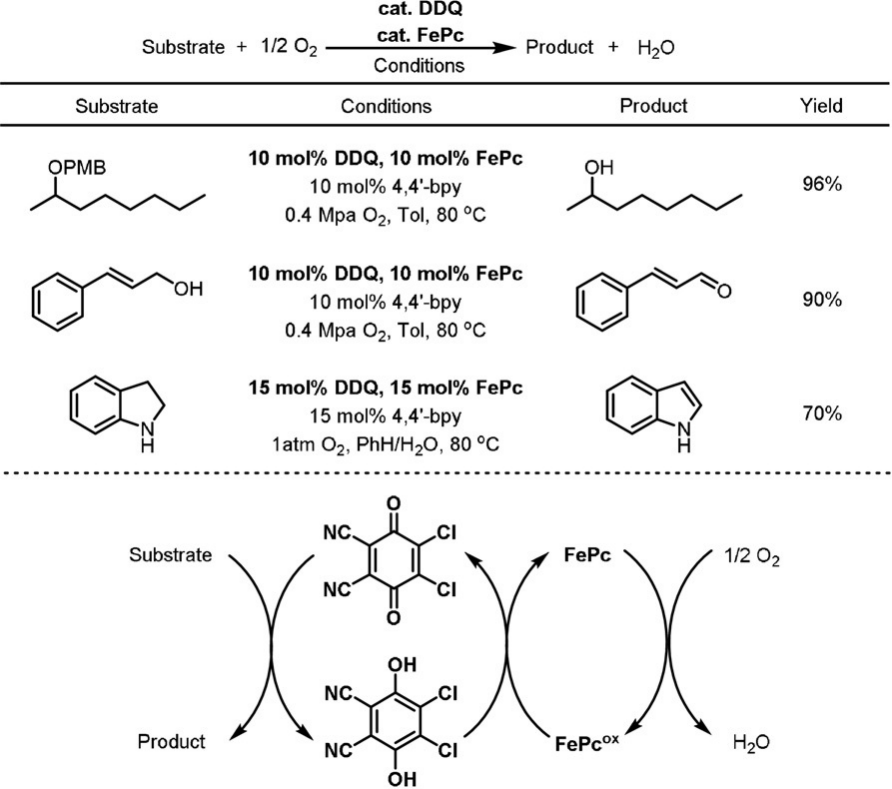
DDQ‐catalyzed aerobic oxidations.

2,3‐Dichloro‐5,6‐dicyano‐1,4‐benzoquinone (DDQ) is a powerful oxidant, which has been applied in many oxidative reactions.^[62]^ Catalytic oxidation systems with DDQ as the catalyst and O_2_ as the terminal oxidant have seen much development in recent years. Hu and Shen reported the use of DDQ as substrate‐selective redox catalyst for the aerobic oxidative deprotection of PMB (*p*‐methoxybenzyl) ether, alcohol oxidation, and aromatization of indoline in high conversions and excellent selectivity (Scheme 20). In this reaction, FePc was employed as the ETM, and O_2_ was used as the environmentally benign terminal oxidant.^[63]^ For the purpose of reusing the FePc employed as ETM, it was supported in multiwalled carbon nanotubes (MWCNTs), which provided good reactivity and recyclability in the aerobic deprotection of PMB ethers.

**Scheme 21:**
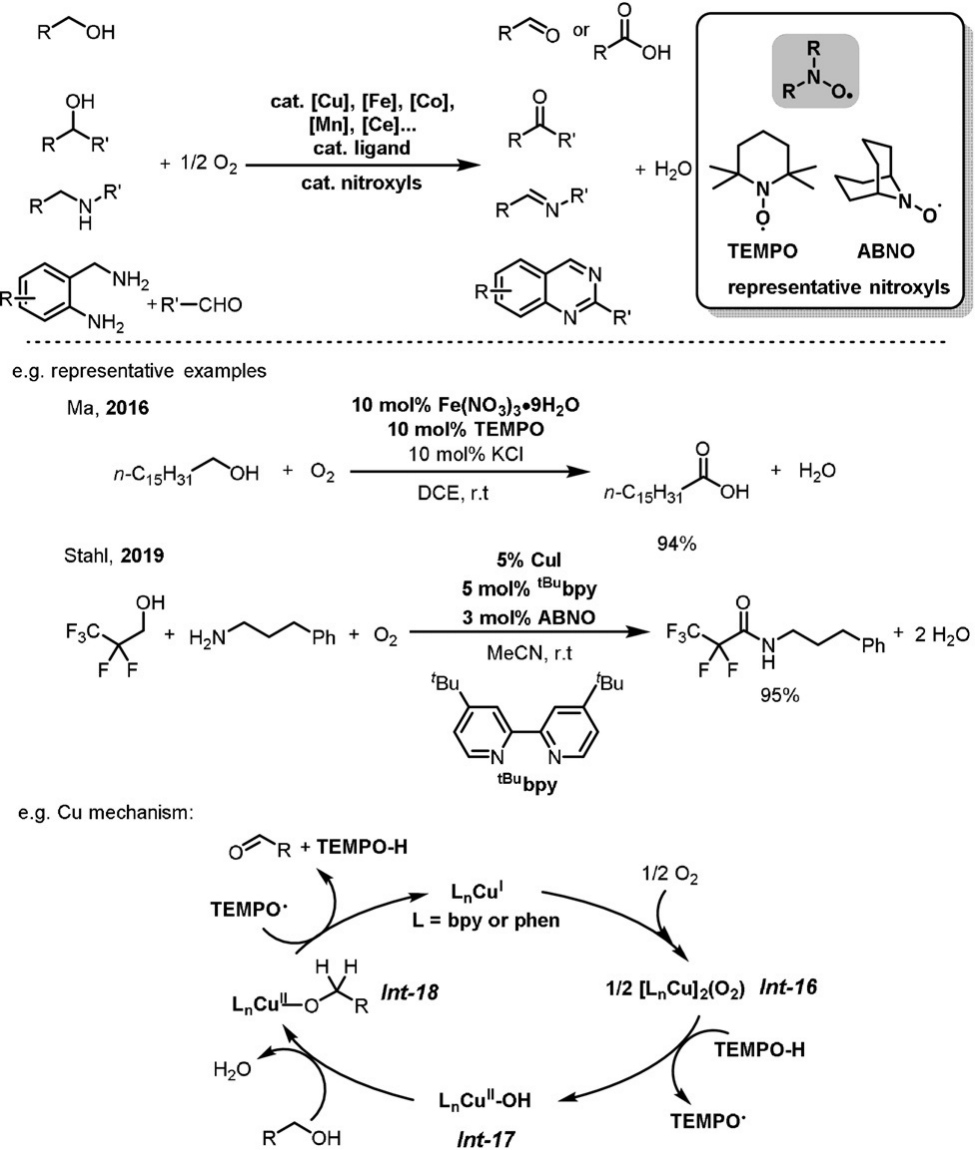
Nitroxyl‐catalyzed aerobic oxidation of alcohols and amines.

Nitroxyl catalysts, such as TEMPO (2,2,6,6‐tetramethylpiperidine *N*‐oxyl) and ABNO (9‐azabicyclo[3.3.1]nonane *N*‐oxyl), have been identified as highly effective catalyst systems for aerobic oxidation (Scheme 21).^[64]^ Various oxidation products can be obtained with the metal/nitroxyl catalyst system under aerobic conditions. For example, Ma and co‐workers reported an Fe(NO_3_)_3_⋅9 H_2_O/TEMPO catalyst system for the selective and mild aerobic oxidation of a wide range of alcohols to carboxylic acids.^[65]^ The Stahl group demonstrated highly practical applications of the Cu/ABNO‐catalyzed aerobic oxidative coupling of alcohols and amines for the synthesis of diverse amides.^[66]^ Homogenous aerobic oxidation with nitroxyl catalysts has also been achieved with other transition‐metal salts (e.g. Mn, Co, and Ce) as co‐catalysts.^[67]^ Among these metals, the Cu/nitroxyl catalyst systems, using nitroxyls such as TEMPO and ABNO, have emerged as some of the most efficient catalysts available for aerobic oxidations. The mechanism and kinetics of Cu/nitroxyl catalysis has been extensively studied by Brackman, Semmelhack, Sheldon, Koskinen, and Stahl in the past years.^[68]^ The simplified catalytic mechanism with the Cu/TEMPO system is depicted in Scheme 21. Initially, aerobic oxidation of Cu^I^ and TEMPOH affords the Cu^II^‐OH species ***Int‐17*** and TEMPO. The oxidation of the alcohol proceeds through the formation of the Cu^II^‐alkoxide species ***Int‐18***, which is then followed by hydrogen atom transfer to TEMPO with formation of the aldehyde and TEMPOH. The Cu/TEMPO catalyst system was extensively studied and applied in the aerobic oxidation of alcohols and amines. An elegant review on this topic was published by Stahl and co‐workers in 2014.^[69]^

**Scheme 22:**
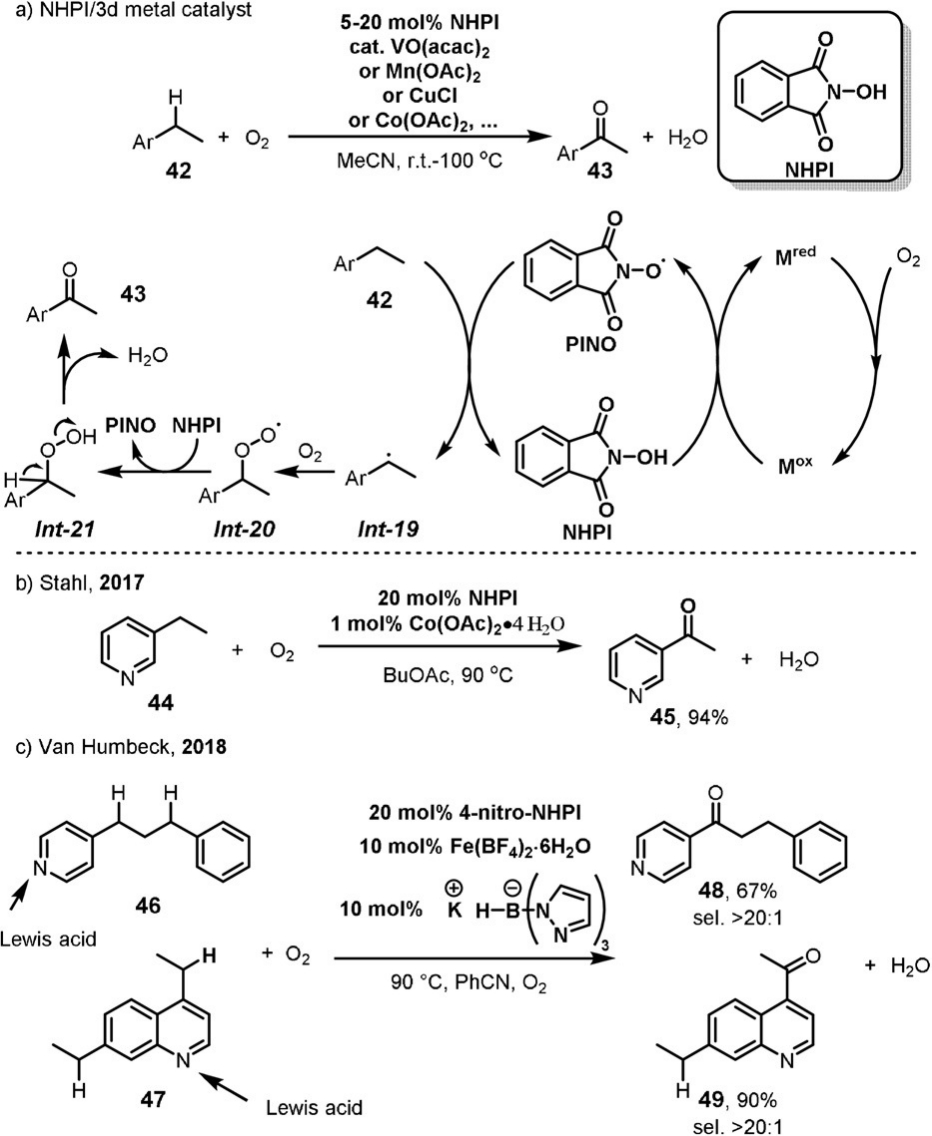
Representative NHPI‐catalyzed of oxidation of C(sp^3^)−H bonds.

*N*‐hydroxyphthalimide (NHPI) is also widely used as a substrate‐selective redox catalyst (SSRC) in selective oxidation reactions. Several 3d transition metals, such as V^4+^, Fe^3+^, Co^2+^, and Cu^2+^ have been utilized as ETM catalysts together with NHPI as SSRC for the oxygenation of C(sp^3^)−H bonds (Scheme 22 a).^[70]^ The reaction initially converts NHPI to the phthalimide N‐oxyl radical (PINO) in the presence of the 3d metal catalyst and O_2_. PINO then mediates the selective H abstraction from the benzylic C−H bond in **42** to generate an organic radical ***Int‐19*** that can react with O_2_. Then H‐atom transfer generates peroxide intermediate ***Int‐21*** and finally produces the oxygenated products **43**, such as ketones or aldehydes and water. Several reviews were published recently on this topic, where the scope and mechanism of these reactions are discussed.^[71]^ Stahl demonstrated an aerobic oxidation of heteroarenes **44** to their corresponding heteroarylketones **45** using NHPI and Co(OAc)_2_⋅4 H_2_O as ETMs (Scheme 22 b).^[72]^ The heterocycles **44** play an important role in this type of oxidation since an enamine or a similar tautomer can often form which is more amenable to oxidation at the benzylic position. In 2018, Van Humbeck reported an interesting related dual‐catalysis approach in which Fe(BF_4_)_2_⋅6 H_2_O, a borate ligand, and a NO_2_‐substituted analogue of NHPI were used in tandem for the aerobic oxidation of substrates bearing an azaheterocycle (**46** and **47**) (Scheme 22 c).^[73]^ The iron complex is introduced to selectively oxidize a benzylic position adjacent to an azaheterocycle because it can act as a Lewis acid, which coordinates to the nitrogen and weakens the C−H bond at the benzylic position, making the hydrogen abstraction and subsequent oxidation easier.

**Scheme 23:**
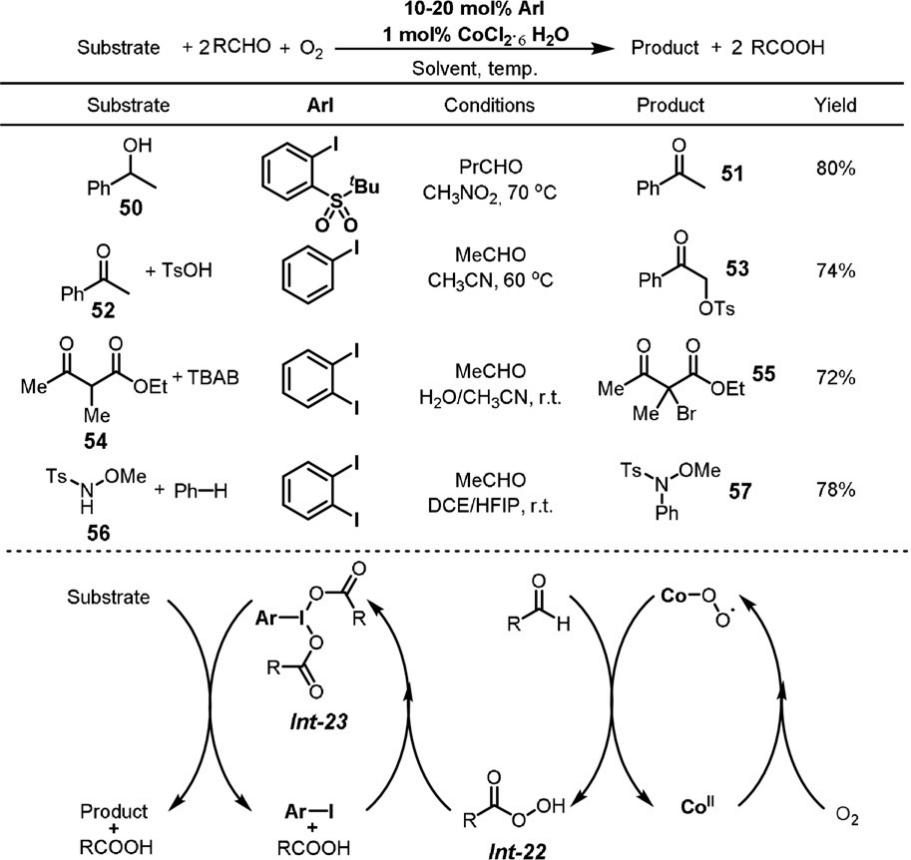
ArI‐catalyzed aerobic functionalizations.

Hypervalent iodine reagents are a useful class of chemical oxidants which find application in diverse chemical syntheses.^[74]^ The current liabilities of hypervalent iodine reagents include the frequent need for stoichiometric amounts of these compounds and their wasteful synthesis using metal‐based oxidants such as KMnO_4_, NaIO_4_, oxone, and *m*CPBA. Very recently, the Powers group reported an efficient formation of hypervalent iodine compounds from aryl iodides, cobalt, aldehydes, and O_2_ (Scheme 23).^[75]^ In this reaction, CoCl_2_⋅6 H_2_O acts as an initiator for aldehyde autoxidation. The aerobically generated peracid ***Int‐22*** (e.g. AcOOH) allows for the oxidation of the aryl iodide to the hypervalent iodine reagent ***Int‐23*** (e.g. PhI(OAc)_2_). With this method, a family of hypervalent iodine reagents were successfully generated in situ using O_2_ as the terminal oxidant. The hypervalent reagents were used in aerobic oxidation in a variety of reactions, such as alcohol oxidation (**50** to **51**), α‐oxygenation of acetophenone (**52** to **53**), bromination of a β‐keto ester (**54** to **55**), and intermolecular C‐H amination (**56** to **57**).

## 3 Metal‐Free Aerobic Oxidations with ETMs

In the aerobic oxidation of organic molecules, many of the processes rely on a metal catalyst or co‐catalyst to facilitate the redox process. However, the presence of trace amounts of metals in the desired products always has several negative effects for biological and medicinal applications. In this context, aerobic oxidations using metal‐free catalytic systems are of great interest in organic synthesis.^[76]^

**Scheme 24:**
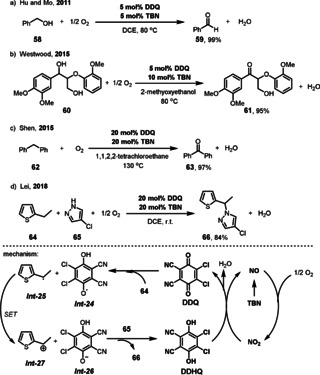
DDQ/TBN catalyst system for aerobic oxidations.

Hu and Mo reported a DDQ/TBN (TBN=*tert*‐butyl nitrite) catalyst system for the selective oxidation of benzylic alcohol **58** to the corresponding aldehyde **59** (Scheme 24).^[77]^ Key to the success for this aerobic oxidation is that the NO released from TBN can act as an ETM between DDQ and molecular oxygen. The same catalyst system can also be applied in the catalytic benzylic oxidation of the lignin β‐O‐4 model **60** to **61** in high yields for efficient biomass conversion and oxygenation of C(sp^3^)−H bonds.^[78]^ Recently, Lei and co‐workers described an oxidative C(sp^3^)–H/N–H cross coupling by introducing DDQ and TBN under aerobic conditions.^[79]^ This amination reaction can be applied to a wide range of alkyl (hetero)arenes **64** and triazoles, pyrazoles and their derivatives **65**. This reaction starts with a hydrogen atom transfer (HAT) process between DDQ and the alkyl (hetero)arene **64**, which then generates the alkyl radical ***Int‐25***. Subsequently a single‐electron oxidation of the alkyl radical ***Int‐25*** leads to the alkyl cation ***Int‐27***, which reacts with the *N*‐nucleophile **65** to furnish the amination product **66**. Meanwhile, DDQH_2_ is oxidized to DDQ by NO_2_, which is generated from TBN and O_2_.

**Scheme 25:**
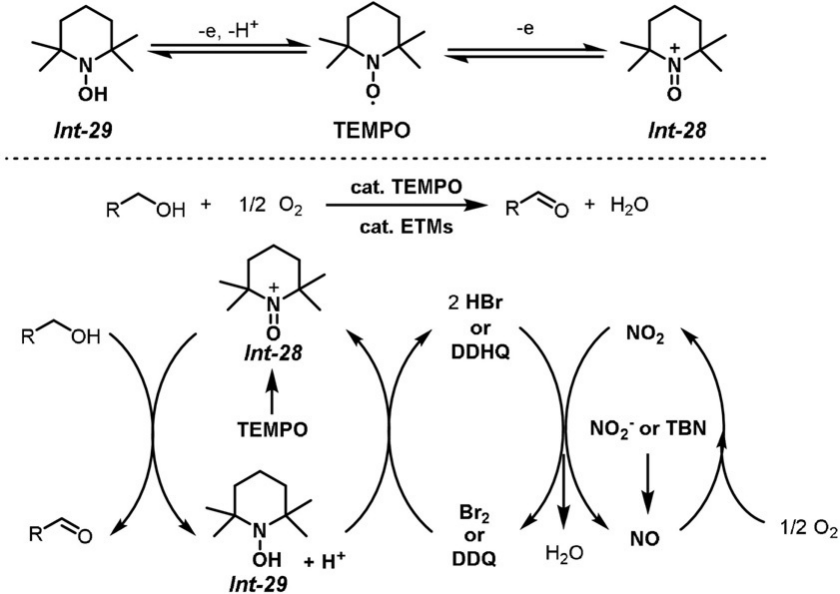
Aerobic oxidation of alcohols with TEMPO as catalyst.

TEMPO is a well‐known, commercially available, and stable nitroxyl radical. One‐electron oxidation of TEMPO affords the oxoammonium species ***Int‐28*** which can be used as an oxidant. For example, oxoammonium‐mediated alcohol oxidation results in two‐electron reduction of the oxoammonium species to afford a hydroxylamine ***Int‐29***. Hu and co‐workers reported a transition‐metal‐free aerobic oxidation of benzylic and heteroaromatic alcohols, with TEMPO as an efficient substrate‐selective redox catalyst (Scheme 25).^[80]^ In this example, Br_2_ and NaNO_2_ are used as the co‐catalysts to relay the electrons between TEMPOH and O_2_. A wide range of primary and secondary alcohols were effectively oxidized. In addition, the use of HBr/TBN or DDQ/TBN instead of Br_2_/NaNO_2_ as ETMs also promotes the aerobic oxidation of alcohols to the corresponding carbonyl compounds in excellent yields.

**Scheme 26:**
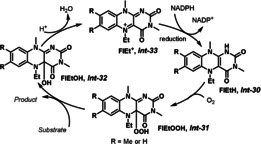
The mechanism of flavin‐catalyzed aerobic oxidations.

It is worth mentioning that flavins, a family of the most versatile redox cofactors in nature, can be used as the substrate‐selective redox catalyst for mild chemical oxidations.^[81]^ The general catalytic cycle is outlined in Scheme 26. The oxidation of the reduced flavin FlEtH ***Int‐30*** with O_2_ occurs readily to give hydroperoxide FlEtOOH ***Int‐31***. Then, this intermediate oxidizes the substrate to the product, generating hydroxyl flavin FIEtOH ***Int‐32***. After elimination of the OH group to give FlEt^+^ ***Int‐33***, a reduction process occurs to regenerate FlEtH ***Int‐30***, and subsequently ***Int‐30*** can be oxidized by O_2_ to ***Int‐31***.

In nature, flavins occurring in monooxygenases use O_2_ as the oxidant. This process requires a cofactor that reduces the hydroxyflavin that is produced after the hydroperoxyflavin has oxidized the substrate, and in nature this cofactor is NADPH (Scheme 26). In an elegant study, Imada, Murahashi, and co‐workers mimicked this process with the development of a flavin‐catalyzed aerobic oxidation of sulfides and tertiary amines at ambient temperature.^[82]^ Hydrazine was used as a stoichiometric reductant and constitutes a mimic of the NADPH cofactor. In addition, other reductants such as metallic zinc,^[83]^ Hantzsch ester,^[84]^ and formic acid^[85]^ were also applied in flavin‐catalyzed aerobic oxidation.

The naturally based flavin derivatives in Scheme 26 involving FlEtOOH (***Int‐31***) have been used as SSRCs in aerobic oxidations, but not so far as ETMs. However, with a related flavin, involving the (*N*,*N*)‐1,3‐dimethyl isomer instead of the FlEtOOH (*N*,*N*)‐3,10‐dimethyl flavin, a hydrogen peroxide‐based biomimetic asymmetric dihydroxylation of olefins was developed, where the flavin and *N*‐methylmorpholine (NMM) act as ETMs with OsO_4_ as the SSRC.^[86]^

**Scheme 27:**
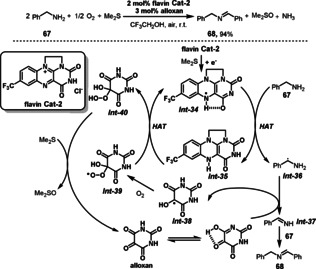
Flavin‐catalyzed aerobic oxidations of amines.

Carbery and co‐workers demonstrated a cationic flavin‐based catalyst system in the biomimetic oxidation of benzylamines to imines (Scheme 27).^[87]^ The combination of a synthetic flavin with alloxan significantly facilitates biomimetic amine oxidation to imines. The mechanism of this aerobic oxidation combines several multistep electron transfer processes. Initially, the flavin catalyst **Cat‐2** is reduced by Me_2_S to generate the neutral semiquinone ***Int‐34***. Subsequently, H‐atom transfer (HAT) takes place between ***Int‐34*** and benzylamine **67** to give semiquinone ***Int‐35*** and α‐amino radical ***Int‐36***. This α‐amino radical ***Int‐36*** can reduce alloxan to α‐hydroxyl carbon radical ***Int‐38*** forming ***Int‐37***. The coupling of ***Int‐37*** with benzylamine **61** leads to the desired product imine **68**. ***Int‐38*** subsequently reacts with O_2_, thus generating peroxyl radical ***Int‐39***. The peroxyl radical ***Int‐39*** oxidizes ***Int‐35*** to ***Int‐34***, to produce hydroperoxide ***Int‐40***. Me_2_S mediates the reduction of ***Int‐40*** to alloxan and formation of stoichiometric DMSO.

## 4 Photo‐ and Electrochemically Mediated Aerobic Oxidations with ETMs

**Scheme 28:**
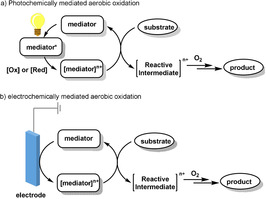
Photo‐ and electrochemically mediated aerobic oxidation reactions.

Photoredox chemistry and electrochemistry have attracted broad interest for the development of green chemical synthesis. These transformations, which employ inexpensive, ubiquitous light and electricity constitute a powerful strategy for the activation of small molecules.^[88]^ Photoredox catalysis offers access to the unique chemical reactivities of organic molecules in the excited state, which allows the generation of reactive intermediates with high redox potential in a transient state. Electrochemistry also offers a solution to this issue by using electric current as the traceless redox agent, and the introduction of a mediated electron transfer can occur against a potential gradient, meaning that lower potentials are needed, reducing the probability of undesired side reactions.^[89]^ In this section, we discuss recent examples in photo‐ and electrochemically mediated aerobic oxidation reactions (Scheme 28).

**Scheme 29:**
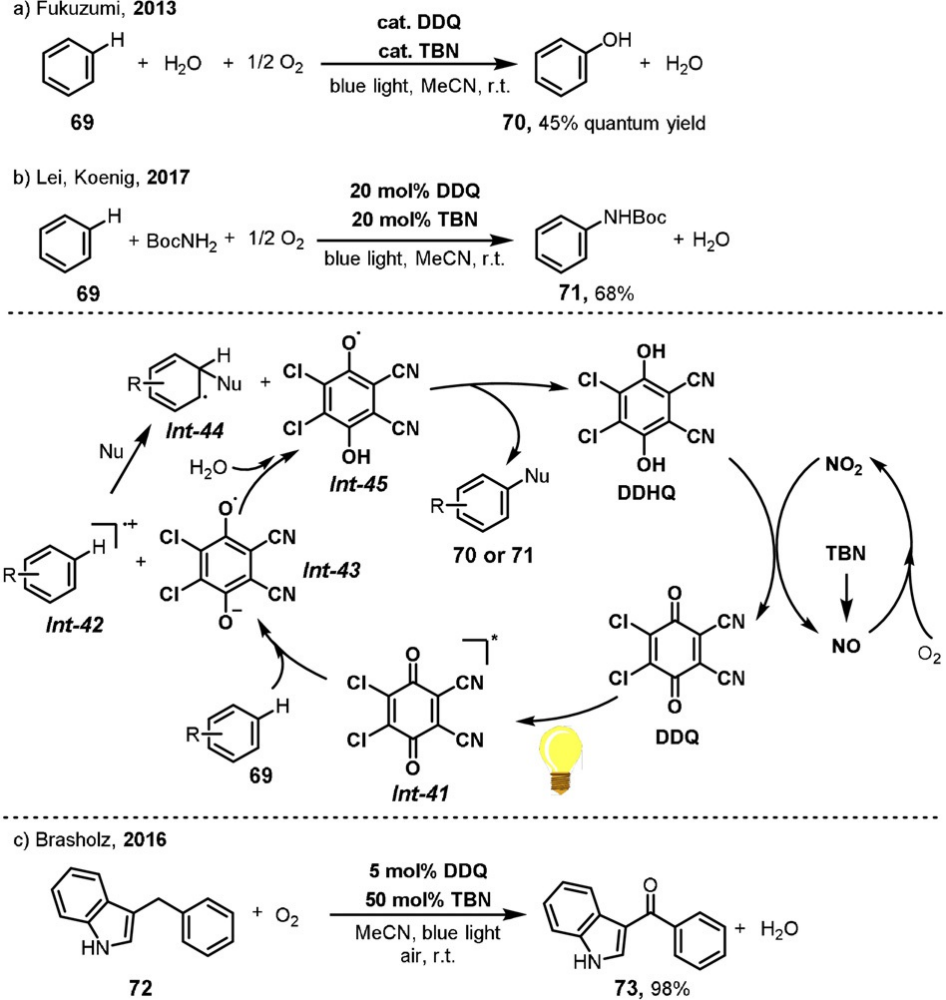
Visible‐light‐mediated DDQ‐ and TBN‐catalyzed functionalizations of arenes and oxygenation of C(sp^3^)−H bonds.

Phenol is an important precursor for many chemicals and industrial products. One‐step oxygenation of benzene to phenol is one of the dream chemical reactions.^[90]^ The Fukuzumi group reported the direct oxygenation of benzene (**69**) to phenol (**70**) under visible‐light irradiation of DDQ under aerobic conditions (Scheme 29 a).^[91]^ This catalyst was also applied in the aerobic amination of aromatics and heteroaromatics by Lei and Koenig (Scheme 29 b).^[92]^ This photooxygenation reaction is initiated by photoinduced electron transfer from benzene (**69**) to the triplet excited state of DDQ ***Int‐41*** to give a benzene radical cation ***Int‐42*** and a DDQ radical anion ***Int‐43***, the protonation of which gives ***Int‐45***. The benzene radical cation ***Int‐42*** then reacts with nucleophiles such as water or amines to yield ***Int‐44. Int‐45*** subsequently reacts with the ***Int‐44*** to form phenol or an aniline derivative, and DDHQ. TBN was used as an ETM to convert DDHQ to DDQ under aerobic conditions. The same catalyst system was also applied for the oxygenation of C(sp^3^)−H bonds in diarylmethane **72** to the corresponding ketones **73** (Scheme 29 c).^[93]^

**Scheme 30:**
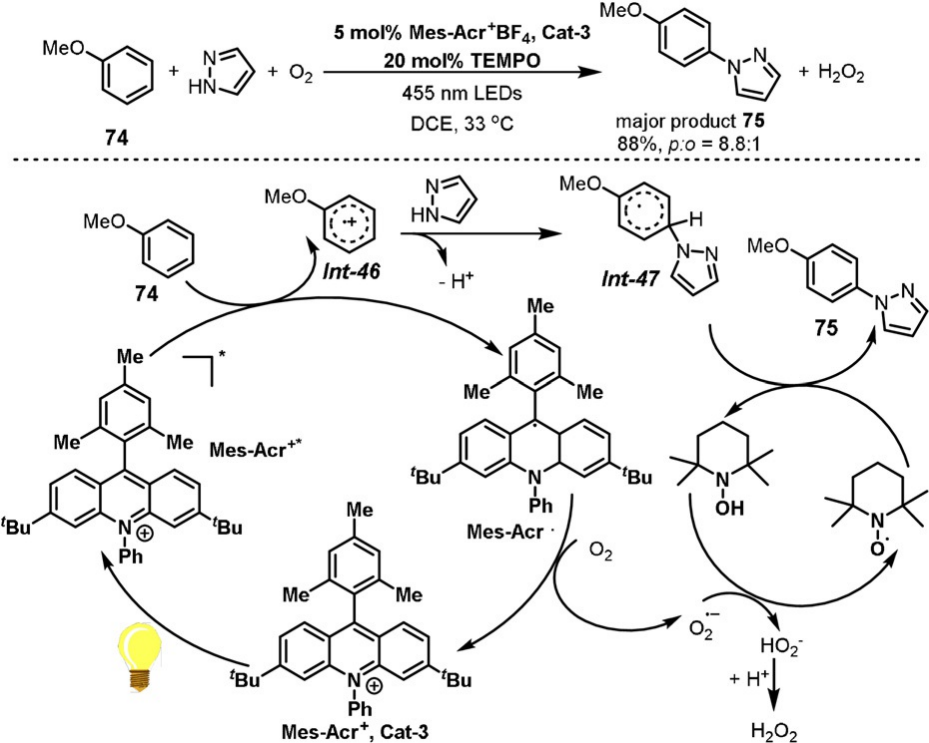
Visible‐light‐mediated acridinium‐ and TEMPO‐catalyzed amination of aromatics.

The Nicewicz group reported an efficient photoredox‐based catalyst system consisting of the photocatalyst Mes‐Acr^+^BF_4_ **Cat‐3**, (Mes, mesityl; Acr, acridinium) and TEMPO for site‐selective amination of a variety of simple and complex aromatics (Scheme 30).^[94]^ The authors proposed that an arene cation radical ***Int‐46*** is generated upon photoinduced electron transfer from the arene to an excited‐state photoredox catalyst (Mes‐Acr^+*^). This arene cation radical ***Int‐46*** could react with imidazole, followed by deprotonation to provide the radical intermediate ***Int‐47***. The subsequent H‐atom transfer with TEMPO would deliver the desired aminated arene **75**. In this catalytic cycle, O_2_ can oxidize acridine radical Mes‐Acr^.^, regenerating acridinium Mes‐Acr^+^ and superoxide O_2_^−.^. The strongly basic superoxide deprotonates TEMPOH via a hydrogen atom transfer process, ultimately forming H_2_O_2_ and regenerating TEMPO.

**Scheme 31:**
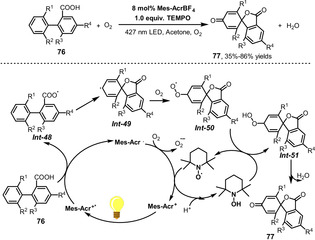
Visible‐light‐mediated dearomatization of biaryls to spirolactones.

Samec and Wang developed an interesting photoinduced dearomatization of nonphenolic biaryl compounds to generate spirolactones (Scheme 31).^[95]^ The dearomatization can be performed via aerobic photocatalysis using Mes‐Acr^+^BF_4_ as the photocatalyst and TEMPO as the ETM. This reaction is induced by electrophilic attack of the carboxyl radical ***Int‐48*** generated from single‐electron transfer with the excited photocatalyst Mes‐Acr^+*^ and substrate **76**. Carboxyl radical ***Int‐48*** then induces dearomatization by intramolecular cyclization to form a spirodiene radical ***Int‐49***, which is then captured by O_2_ to form ***Int‐50*** in aerobic systems, after which H‐atom transfer generates peroxide intermediate ***Int‐51*** and finally spirodienone **77** is produced through the release of water. This method represents a novel route to synthesize spirolactones from the biaryl motif.

**Scheme 32:**
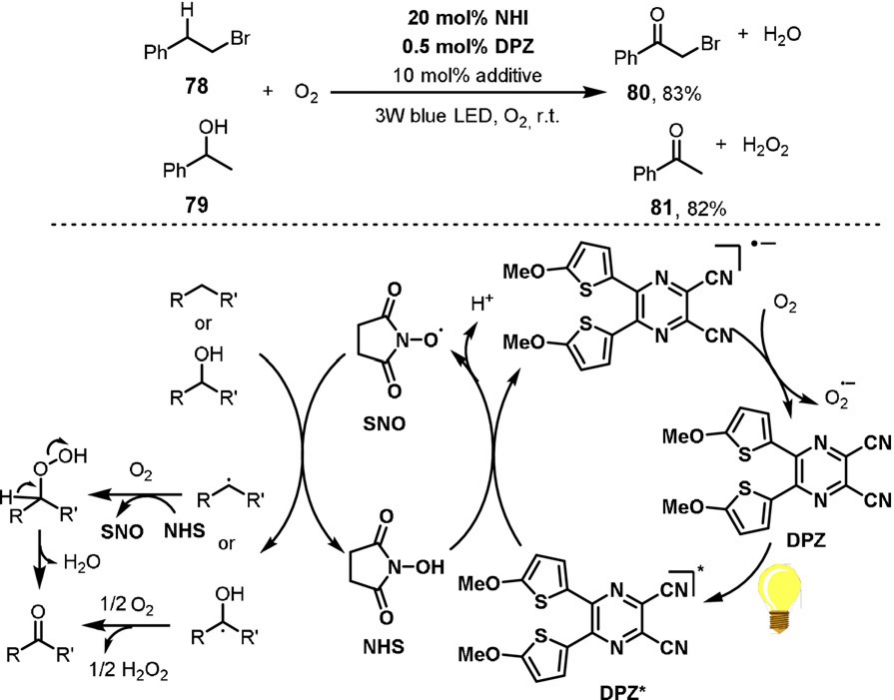
Visible‐light‐mediated benzylic oxygenations.

Jiang and co‐workers demonstrated a benzylic oxygenation using a novel photocatalyst, a dicyanopyrazine (DPZ) derivative in combination with catalytic amounts of *N*‐hydroxysuccinimide (NHS) (Scheme 32).^[96]^ In the presence of light, the photocatalyst DPZ is activated to an excited state DPZ*, which can oxidize NHS in a single‐electron‐transfer process. The succinimide‐N‐oxyl (SNO) radical then abstracts the H atom at the benzylic C–H position of the substrate **78** or **79** to generate a benzylic radical that reacts with O_2_, finally delivering the ketone **80** or **81**.

**Scheme 33:**
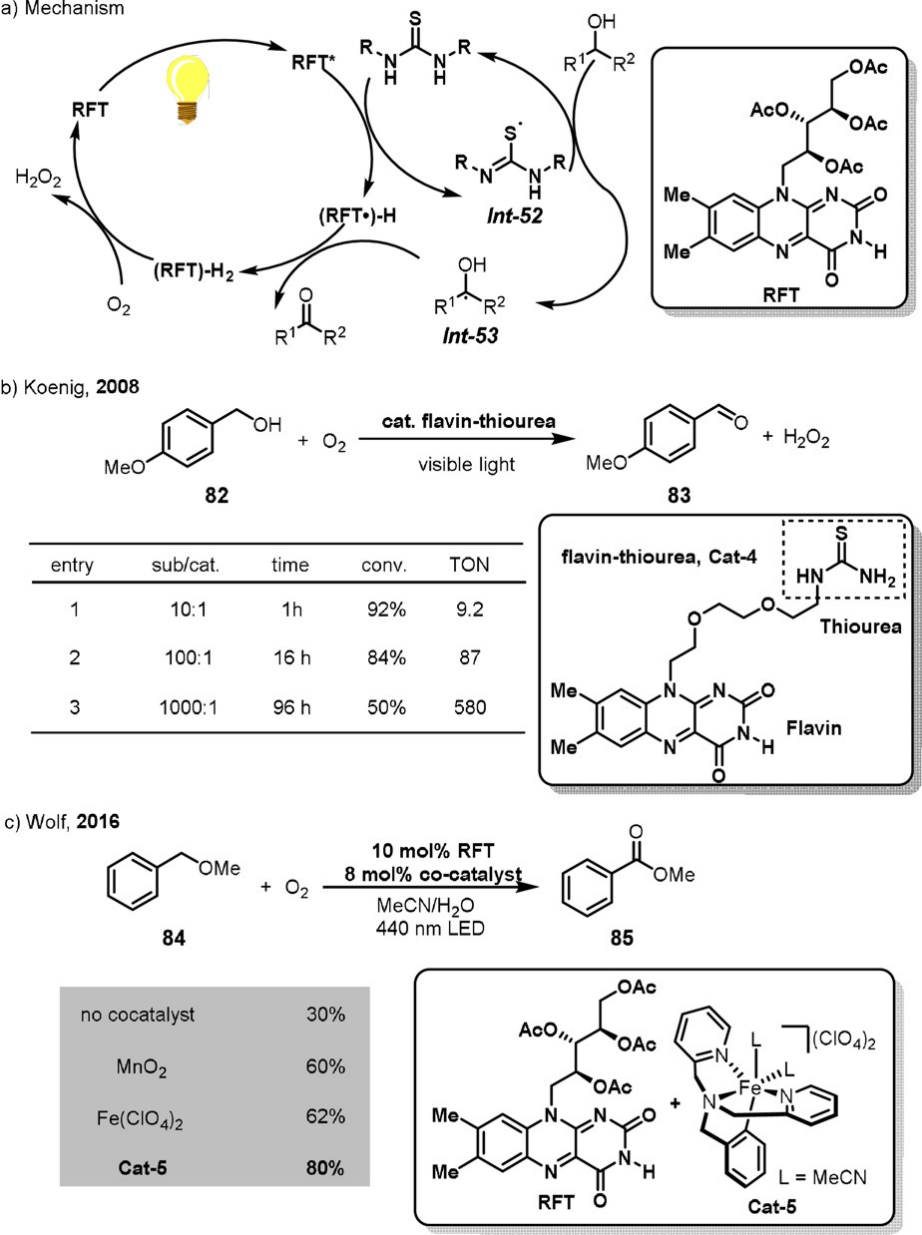
The mechanism and the examples of visible‐light‐mediated flavin‐catalyzed aerobic oxidations.

Flavins have been extensively studied as organic photoredox catalysts for oxidative organic transformations.^[97]^ For example, riboflavin tetraacetate (RFT) is a readily available compound with an oxidation potential of +1.67 V (vs. SCE) upon irradiation (*λ*_max_=440 nm). This characteristic allows RFT and its derivatives to function as photocatalysts in aerobic oxidations and the mechanism is depicted in Scheme 33 a. Upon irradiation, electron transfer followed by proton transfer between excited RFT* and urea results in thiyl radical ***Int‐52*** and semiquinone form (RFT^.^)‐H. The thiyl radical ***Int‐52*** abstracts the α‐hydrogen atom from the alcohol to produce α‐hydroxyl carbon radical ***Int‐53***, which can further react with (RFT^.^)‐H by hydrogen‐atom transfer to afford the desired product ketone. Finally, the reduced species (RFT)‐H_2_ is oxidized by O_2_ to regenerate the RFT catalyst and H_2_O_2_. Based on the interaction of RFT and urea, Koenig designed an efficient bifunctional catalyst consisting of a flavin backbone and a thiourea group (Scheme 33 b).^[98]^ This catalyst is able to catalyze the aerobic oxidation of 4‐methoxybenzyl alcohol (**82**) to the corresponding aldehyde **83** in low catalyst loading (TON=580).

The formation of H_2_O_2_ as a by‐product is a major drawback of the RFT‐catalyzed photocycle because H_2_O_2_ can degrade RFT under irradiation which leads to the corresponding ketones being produced in poor yields. Wolf reported that the addition of an iron complex can significantly improve the yield under aerobic conditions because the iron complex can catalyze H_2_O_2_ disproportionation (Scheme 33 c). The combination of the RFT and the biomimetic non‐heme iron complex Fe(TPA)(MeCN)_2_](ClO_4_)_2_ **Cat‐5** (TPA=tris(2‐pyridylmethyl)amine), gave the best yield of **85** from **84** under visible‐light irradiation and aerobic conditions.^[99]^

**Scheme 34:**
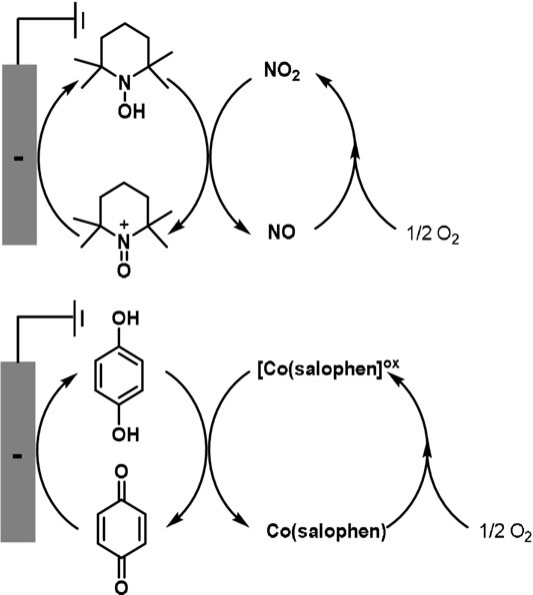
Electrochemical O_2_ reduction with ETMs.

Many catalytic aerobic reactions operate with two half‐reactions: 1) oxidation of an organic molecule, and 2) reduction of O_2_ to water. Efficient reduction of O_2_ to water is a central challenge in many aerobic oxidation reactions as well as in energy conversions.^[100]^ The electrochemical oxygen reduction reaction (ORR) can be achieved by using electron‐transfer mediators to promote efficiency and selectivity. Recently, the Stahl group demonstrated that by combining nitroxyls (such as TEMPO) with NO_x_, it is possible to achieve efficient electrocatalytic O_2_ reduction at high potentials (Scheme 34).^[101]^ In the coupled redox reactions, the nitrogen oxide catalyst drives aerobic oxidation of a nitroxyl mediator to an oxoammonium species, which is then reduced back to the nitroxyl at the cathode. The same group also explored a molecular cobalt complex, Co(salophen), and HQ as ETMs for the electrochemical reduction of O_2_ to water.^[102]^ They showed that redox cooperativity between Co(salophen) and HQ enables O_2_ reduction at higher potentials and with faster rates than those observed with either individual catalyst partner. These coupled catalyst systems with ETMs demonstrate a unique strategy to achieve improved performance in electrochemical ORR.

**Scheme 35:**
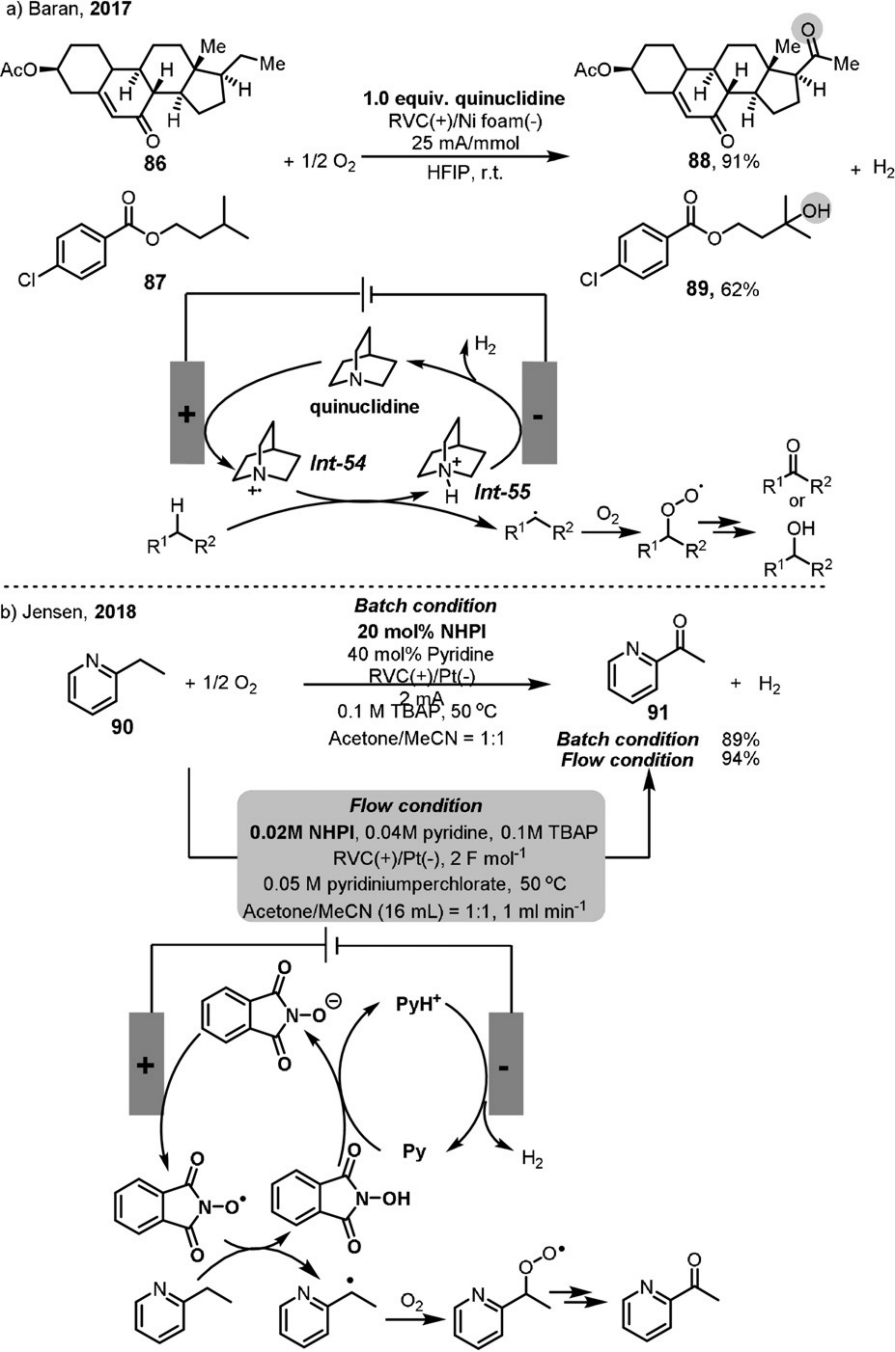
Electrooxidation of C(sp^3^)−H bond to ketones and alcohols.

It is widely recognized that simple aliphatic C−H bonds are more difficult to oxidize and selectivity is more difficult to achieve compared with activated C(sp^3^)−H bonds (benzylic or allylic).^[103]^ Recently, the Baran group reported an efficient aerobic electrochemical oxidation of unactivated C−H bonds (in **86** and **87**) to the corresponding ketones or alcohols (**88** and **89**) (Scheme 35 a).^[104]^ Inspired by their previous work on the anodic allylic C‐H oxidation,^[105]^ in this reaction, the use of one equivalent of quinuclidine as an electrochemical mediator allows efficient electrooxidation of C−H bonds. Mechanistically, the electrochemical C‐H oxidation involves a quinuclidine radical cation ***Int‐54*** generated through anodic oxidation. This high‐energy species can homolytically cleave an unactivated C−H bond. Then a reaction between the carbon‐centered radical and O_2_ affords the oxidation product. This method has the potential to facilitate the synthesis of complex molecules in good yields with high selectivity. Jensen presented a continuous electrolysis system engineered for NHPI‐mediated electrochemical aerobic oxidation of benzylic C−H bonds (Scheme 35 b).^[106]^ Here, NHPI and pyridine were used as efficient mediators for electron transfer. The deprotonation of NHPI by pyridine, followed by anodic electron transfer, leads to a PINO radical. The PINO radical subsequently mediates abstraction of a benzylic hydrogen atom in **90** to generate a benzylic radical. This radical can be trapped by O_2_ to form a peroxy radical, which then yields the carbonyl products **91**. The corresponding cathode reaction is the reduction of pyridinium cation to evolve H_2_ and regenerate pyridine. The use of an electrochemical flow system for aerobic oxidation enables a process for further scaling‐up and opens up potential for an industrial process.

## 5 Conclusion and Outlook

Aerobic oxidation reactions are of fundamental importance in chemical science and are widely applied in synthetic organic chemistry. In this Minireview, recent progress made in aerobic oxidations using electron transfer mediators has been summarized and discussed. The coupled catalytic system significantly facilitates the transport of electrons from the reduced substrate‐selective catalyst to O_2_, thereby increasing the efficiency of aerobic oxidation. This strategy has proven to be powerful and valuable in the assembly of carbon–carbon and carbon–heteroatom bonds, which provides useful applications in homogenous catalysis and organic synthesis.

Despite the advances reported in recent years, we feel that many exciting opportunities and challenges still lie ahead in the field of aerobic oxidative transformations:

1. The design of novel and efficient ETMs is highly important and desirable for aerobic oxidations. For example, the use of chiral ETMs for stereoselective synthesis can be explored and that deserves more attention. Additionally, new concepts like solid‐supported mediators can be developed in order to improve the efficiency of aerobic processes and simplify product isolation. Computational methods can also be routinely used to assist in tailoring the properties of redox catalysts to meet specific purposes.
2. The use of first‐row transition metals as substrate‐selective redox catalysts (SSRCs) is one of the key developments in aerobic oxidations, due to the abundance, low price, and low toxicity of these metals.
3. The recent progress in photo‐ and electrochemical reactions in aerobic oxidations will attract broad interest for the development of green chemical synthesis. These transformations, which use inexpensive, ubiquitous visible light and electric current constitute a powerful strategy for the activation of small molecules.

Clearly, the field of mediated electron transfer in aerobic oxidation constitutes a fertile arena in which to conduct research. We have no doubt that many important and exciting developments will be forthcoming in the future.

As mentioned in the introduction, large‐scale industrial applications with molecular oxygen at high pressure require rigorous precautions. The advantage of many of the biomimetic aerobic oxidations described in this Minireview is that they can often be run under mild conditions at ambient pressure. Furthermore, dilute concentrations of O_2_ as in air or <5 % O_2_ in N_2_ can also be used in many cases. Under the latter conditions the safety problems are dramatically reduced.

## 6 Addendum

After the submission of the present article, two papers related to its content on aerobic oxidation have been published by Stahl and co‐workers.^[107]^ The first paper^[107a]^ describes the Pd‐catalyzed aerobic homocoupling of thiophenes using phenanthroline dione and a Cu(OAc)_2_ as electron transfer mediators (ETMs). In the second article^[107b]^ involving Pd‐catalyzed aerobic C‐H arylation it was found that tailored quinones such as 2,5‐di‐*tert*‐butyl‐*p*‐benzoquinone led to high turnovers (>1900) on Pd.

## Conflict of interest

The authors declare no conflict of interest.

## Biographical Information

*Jie Liu was born in 1990 in Hunan (China). He studied at Wuhan University, where he obtained his BS and MSc degrees in 2012 and 2014 under the supervision of Prof. Aiwen Lei. Then he joined Prof. Matthias Beller's group at the Leibniz Institute for Catalysis (Germany), where he received his PhD degree in 2017. Following postdoctoral work with Prof. Jan‐E. Bäckvall at Stockholm University, he became a principal investigator in department of chemistry at Hunan University in 2020*.

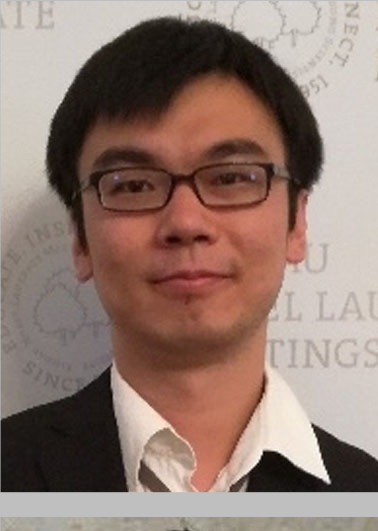

## Biographical Information

*Arnar Guðmundsson was born in 1990 in Reykjavík (Iceland). He studied at the University of Iceland, where he obtained his BS degree in 2012. Later he pursued his MSc degree at Stockholm University under the supervision of Prof. Jan‐E. Bäckvall and later joined his group as a PhD student in 2016*.

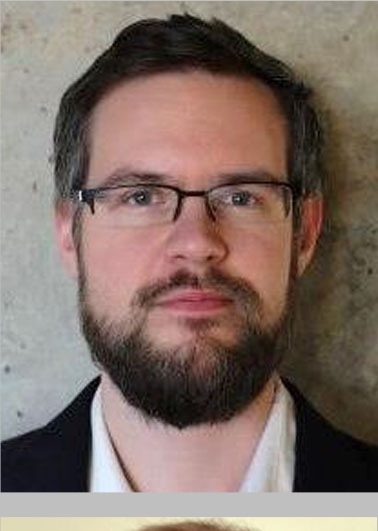

## Biographical Information

*Jan‐Erling Bäckvall was born in Malung (Sweden) in 1947. He received his PhD from the Royal Institute of Technology, Stockholm, in 1975 with Prof. B. Åkermark. After postdoctoral work (1975–1976) with Prof. K. B. Sharpless, he joined the faculty at Royal Institute of Technology. He was appointed professor of organic chemistry at Uppsala University in 1986. In 1997, he moved to Stockholm University, where he is currently a professor of organic chemistry. His current research interests include transition‐metal‐catalyzed organic transformations, biomimetic oxidations, and enzyme catalysis*.